# Carbon budget of common urban tree species in an arid city based on life cycle assessment

**DOI:** 10.1371/journal.pone.0345213

**Published:** 2026-04-24

**Authors:** Ruijing Zhang, Li Wang, Xiaoge Tian, Yuyang Song

**Affiliations:** 1 Agricultural college, Shihezi university, Shihezi, China; 2 Shihezi Garden Research Institute, Shihezi, China; University of Emergency Management, CHINA

## Abstract

Urban trees sequester and store carbon through photosynthesis, while their planting and maintenance processes consume energy and materials, resulting in the release of carbon back into the atmosphere. Therefore, a comprehensive and scientifically rigorous assessment of the net carbon sequestration capacity of landscape trees must account for both carbon uptake and emissions. In this study, three types of urban green spaces—parks, roadsides, and residential areas—were selected as research sites. Based on the life cycle assessment, biomass equation method and emission factor method, the life cycle carbon sequestration and carbon emissions of the main tree species in Shihezi City were calculated. The genotype main effect plus genotype-by-environment interaction (GGE) biplot and Pearson correlation analyses were employed to elucidate species–environment interactions across the three green space types and to identify management strategies for maximizing net carbon sequestration. The main findings were as follows: (1) The mean carbon storage per tree in parks (366.78 kgC tree^-1^) was significantly higher than that in roadsides (305.59 kgC tree^-1^) and residential green spaces (236.39 kgC tree^-1^), with *Ulmus pumila* exhibiting the highest per-tree carbon storage (595.39 kgC tree^-1^). (2) The order of carbon emissions in the whole life cycle is: park (461.15 kgC tree^-1^)> roadside (395.48 kgC tree^-1^)> residential area (283.65 kgC tree^-1^). Irrigation is the main emission source, accounting for more than 50% of the total carbon emissions in the whole life cycle. (3) Under current management practices, *Ulmus pumila* consistently maintained a positive life cycle carbon budget across all green space types, whereas some other large tree species showed positive carbon budgets only in specific green space types and had negative carbon budget in others. All small tree species exerted negative effects on the carbon sequestration function of urban green spaces across all three types of green space. Based on the findings of this study, strategies for low-carbon urban green space construction were proposed through enhancing carbon sequestration and reducing maintenance-related emissions.

## Introduction

Against the backdrop of accelerating global climate change, cities—being the most intensive systems of human activity—are facing unprecedented ecological and climatic challenges [1]. In response, many municipal governments have implemented policies to promoting tree planting, protect urban green spaces, and more recently green buildings [2]. Urban green spaces provide essential ecological services that support the urban environment [3], including mitigating the urban heat island effect [4], reducing air pollution [5], and improving the urban microclimate [6]. Nevertheless, unlike natural forests and other unmanaged vegetation, urban greenery is typically established, cultivated, and intensively maintained by humans. Such maintenance activities often involve the use of fossil fuel-powered equipment—including lawn mowers, chainsaws, and trucks—along with irrigation, fertilization, and material transport, all of which generate considerable CO₂ emissions [7]. Assessing only carbon sequestration or only carbon emissions from urban green spaces presents an incomplete picture. The development of green spaces is a long-term dynamic process, and stock-based assessments tend to overestimate their carbon sink potential by neglecting the emissions generated during management. If carbon emissions from anthropogenic management exceed the sequestration capacity of vegetation, urban landscaping plants may transition from functioning as net carbon sinks to net carbon sources, thereby diminishing the overall carbon sequestration potential of urban vegetation and affecting the urban carbon balance [8,9].

Urbanization is an inevitable process in social and economic development. Like many developing countries, China is experiencing rapid urban expansion, with its urbanization rate projected to exceed 70% by 2050 [10]. In recent years, studies have attempted to quantify carbon budgets at the scale of cities and green spaces. Tang [11] studied the landscape of the second section of Guanggu Road in Wuhan. During the 30-year evaluation period, a total of 558.6 t CO_2_ was emitted during the construction and later maintenance management stages, while the total amount of CO_2_ absorbed by trees and shrubs was only 433.2 t. Park and Jo [12] conducted a 30-year life cycle carbon budget assessment of 30 small- and medium-sized parks in Korea, repoerting that although trees and shrubs accumulated 17.8 kg·m ⁻ ² of carbon over three decades, greenhouse gas emissions from seedling production, paving materials, irrigation, pruning, lawn mowing, and waste disposal reached 9.3 kg·m ⁻ ². Similarly, Jo [13] reported that in a residential green space in Chicago, annual carbon emissions from maintenance and deadwood decomposition/disposal accounted for approximately 58–65% of the site’s annual carbon uptake. Collectively, these studies indicate that even in regions with relatively abundant precipitation, carbon emissions associated with maintenance can substantially offset, and in some cases entirely negate, the carbon sequestration benefits of urban trees. Although research on urban green space carbon budget has increased in recent years, most studies have been conducted in regions with different climatic and environmental settings. Variations in evaluation indicators, analytical methods, and plant species further complicate cross-study comparisons. Even for the same tree species, methodological discrepancies often lead to inconsistent results, hindering the establishment of a unified framework for evaluating urban carbon budgets and limiting the practical applicability of these findings to tree species selection and management. In arid areas, precipitation is typically scarce and highly variable between years, evapotranspiration rates are high, and soil water holding capacity is limited [14]. To maintain tree survival and ensure their landscape and ecological functions, more frequent and intensive irrigation, along with more precise plant protection measures, are often required [15]. These conditions raises a critical question: do urban trees in arid regions genuinely function as carbon sinks? To maximize the carbon sequestration potential of urban green spaces, it is essential to scientifically assess the carbon storage of trees and the carbon emissions from their maintenance across different green space types, thereby clarifying their actual role in the urban carbon cycle. Such assessments are of great significance for achieving urban carbon balance goals.

## Materials and methods

### Overview of the study area

Shihezi City (43°26′ ~ 45°20′N, 84°58′ ~ 86°24′E) is located in the northern part of the Xinjiang Uygur Autonomous Region, situated on the flat terrain of the northern foothills of the Tianshan Mountains. The administrative area covers approximately 460 km^2^. The city experiences a typical temperate continental arid climate, with an annual average temperature is 7.1 ~ 8.7 °C, annual precipitation ranging from 64.1 ~ 222.9 mm, annual sunshine duration of 2454.4 ~ 2718.9 h, and a frost-free period of approximately 180 days. Currently, Shihezi City has a total green space area of 2680 ha, including 341 ha of public green space, with a green coverage rate in the built-up area of 43%, placing it among the leading cities in Xinjiang in terms of urban greening.

### Research material

Field surveys were conducted to identify fourteen high-frequency backbone tree species occurring in parks, roadside green spaces, and residential areas in Shihezi, which were selected as the focus of this study. Including *Fraxinus rhynchophylla*, *Fraxinus chinensis*, *Ulmus laevis*, *Ulmus pumila*, *Salix babylonica*, *Quercus robur*, *Pinus sylvestris*, *Robinia pseudoacacia*, *Acer negundo*, *Rhus typhina*, *Syringa reticulata*, *Crataegus pinnatifida*, *Amygdalus davidiana*, *Malus spectabilis*. Due to the influence of urban planning, maintenance and management, trees will not complete the whole growth and development process in urban green space, and there are certain application years. Based on expert questionnaire survey and field investigation, this study comprehensively considered the factors such as tree life, growth rate and renewal frequency in urban green space. Finally, the general application years of different tree species in urban green space were set as follows: 30 years for small trees and 50 years for trees. In order to ensure the comparability between the research objects, we selected the plants with normal growth and good health from the above 14 tree species in each green space type.

### Estimation of plant biomass equation in urban green space

The calculation of carbon storage in urban green space is divided into two parts: aboveground and underground. This study mainly focuses on the tree layer, and does not calculate the carbon storage of shrubs, ground cover and soil. To obtain robust allometric equations, we collected multi-age samples covering juvenile to mature stages for each species, comprising 10 age classes. In each age class, representative trees were selected across three types of urban green spaces, yielding about 30 trees per species in total (S1 Table). For each sampled tree, aboveground biomass was estimated using a non-destructive sectional volume measurement approach. Diameter at breast height (DBH, measured at 1.3 m) was recorded with a diameter ruler, and tree height was measured with an altimeter (ECII D-R). A Spiegel Relaskop was used to record the diameters and lengths of trunk sections and all branches > 5 cm in diameter. Volumes of these components were then calculated using the National Standard Log Volume Tables (GB/T 4814–2013) and multiplied by species-specific basic wood density to estimate stem and coarse branch biomass [16]. For branches ≤ 5 cm in diameter, 15 standard branches per species were sampled, oven-dried, and weighed to determine mean dry mass, which was then multiplied by the total number of fine branches per tree to estimate their biomass contribution. Belowground biomass was estimated using root-to-shoot ratios recommended by the IPCC [17]. By combining these components, we obtained whole-tree biomass estimates for each sampled tree. Based on these data, species-specific biomass equations were established using DBH and tree height as independent variables.

*Fraxinus rhynchophylla: W* = 0.153(*D*^2^*H*)^0.921^、*Fraxinus chinensis: W* = 0.389(*D*^2^*H*)^0.810^、*Ulmus laevis: W* = 0.088(*D*^2^*H*)^0.911^、*Quercus robur: W* = 0.282(*D*^2^*H*)^0.818^、*Ulmus pumila: W* = 0.101(*D*^2^*H*)^0.935^、*Pinus sylvestris: W* = 0.322(*D*^2^*H*)^0.798^、*Salix babylonica: W* = 0.105(*D*^2^*H*)^0.928^、*Robinia pseudoacacia: W* = 0.163(*D*^2^*H*)^0.877^、*Acer negundo: W* = 0.499(*D*^2^*H*)^0.774^、*Rhus typhina: W* = 0.086(*D*^2^*H*)^0.961^、*Syringa reticulata: W* = 0.262(*D*^2^*H*)^0.882^、*Crataegus pinnatifida: W* = 0.359(*D*^2^*H*)^0.824^、*Amygdalus davidiana: W* = 0.065(*D*^2^*H*)^1.008^、*Malus spectabilis: W* = 0.627(*D*^2^*H*)^0.734^. where *W* is total biomass (kg), *D* is *DBH* (cm), and *H* is tree height (m). Detailed model diagnostics (sample size, R², RMSE, parameter standard errors) are provided in Supplementary S2 Table. Finally, the total biomass of each species was multiplied by its carbon fraction to obtain the carbon storage of the trees [18], the formula is:


CS=B×CF (1)

In the formula, CS is the carbon storage; B is biomass; and CF is the carbon fraction of the dry biomass, with a default value of 0.5 [19].

### Determination of environmental factors

From July to September 2024, soil samples were taken by the five-point method of plum blossom in different green space types. Five sampling points were selected in the center and four corners of each 5m × 5m small sample square. After removing the gravel and litter on the surface, soil samples were collected at three levels of 0 ~ 10 cm, 10 ~ 20 cm and 20 ~ 30 cm. A total of 90 soil samples were collected from each of the three green space types, which were brought back to the laboratory wind, removed debris, ground and sieved for the determination of soil organic carbon content (potassium dichromate external heating method); the temperature of a single tree (1.5 m from the ground) in the sun and shade was monitored in real time by using an electronic temperature and humidity meter. Soil compactness was measured by TJSD-750 soil compactness meter.

### Full life cycle carbon emissions

This study uses a life cycle assessment (LCA) framework to assess carbon emissions associated with urban green space, including all major stages of tree establishment, growth and removal. The data of each stage were obtained through in-depth interviews with landscaping maintenance personnel and management personnel, supplemented by urban green space maintenance records. The life cycle inventory comprised material and energy inputs, emission factors, and corresponding references (S1 File). To ensure consistency and comparability across life cycle stages, carbon emission conversion factors recommended in the IPCC Guidelines for National Greenhouse Gas Inventories (Table 1) were applied to calculate emissions for each process. Carbon emissions from all life cycle stages were then integrated with carbon sequestration estimates to quantify the total life cycle carbon budget of urban trees. The life cycle carbon emissions of urban trees were calculated using the following formula:

**Table 1:** CO_2_ emission factor coefficients.

Serial Number	Material type	Emission coefficient	Units	Reference source
1	Gasoline	2.03	KgCO_2_e L^-1^	IPCC (2006) [20]
2	Diesel fuel	2.17	KgCO_2_e L^-1^	IPCC (2006) [20]
3	Irrigation	1.85	KgCO_2_e m^-3^	China Product GHG EF Database (2022) [21]
4	Pesticide	18.70	KgCO_2_e kg^-1^	Lal (2004) [22]
5	Germicide	14.30	KgCO_2_e kg^-1^	Lal (2004) [22]


$$CE=\sum_{i=1}^{n}{CE}_{i}\times 12/44=\sum_{i=1}^{n}{\left(m\beta \right)}_{i}\times 12/44$$ (2)

Where *CE* represents the carbon input (KgC); *n* denotes the specific types of energy consumed and material inputs in maintenance management of urban green space plants; *m* is the energy consumption and material input; *β* is the carbon emission coefficient of energy consumption and material input. In addition, the amount of CO_2_ and carbon emission (C) are convertible, and the emission of 1 kgCO_2_ is equivalent to an emission of approximately 0.272 (= 12/44) kgC.

### Estimation of plant carbon budget in urban green space

The carbon budget of major urban green space tree species was calculated as the difference between plant carbon sequestration and carbon emissions. A positive value indicates that the plant functions as a carbon sink, whereas a negative value indicates that it is a carbon source. The carbon budget reflects the plant’s carbon balance status, which is determined by comparing the sequestration value with the emission value; when the sequestration equals the emissions, the plant is considered to be in a relatively balanced carbon budget state. The formula for calculating the life cycle carbon budget (C) of major urban tree species is as follows:


C=CS−CE (3)

Where *CS* represents the total carbon sequestration over the life cycle, and *CE* represents the total carbon emissions over the life cycle. Both are expressed per tree in units of kilograms of carbon (kgC tree^-1^).

### GGE biplot

The GGE (Genotype plus Genotype-by-Environment) biplot, based on environment-centered data, was employed to evaluate species effects and their interactions with the environment. This method emphasizes the genotype (G) main effect and genotype-by-environment interaction (G × E) effect, allowing simultaneous visualization of both components [23], The mathematical model is expressed as:


$$y_{ij}-u-b_{i}=\lambda_{1}\gamma_{j1}{\overset{↼}{\delta }}_{i1}+\lambda_{2}\gamma_{j2}{\overset{↼}{\delta }}_{i2}+\varepsilon_{ij}$$ (4)

Where $y_{ij}$ is the mean carbon budget of the *j*^*-th*^ plant species in the *i*^*-th*^ green space; *u* is the overall mean carbon budget across all plants; $b_{i}$ is the mean carbon budget of all plant species in the *i*^*-th*^ green space; $\lambda_{1}$ and $\lambda_{2}$ are the eigenvalues of the first and second principal components, respectively; $\gamma_{j1}$ and $\gamma_{j2}$ are the eigenvectors of the *j*^*-th*^ plant species on the first and second principal components, respectively; $\delta_{i1}$ and $\delta_{i2}$ are the eigenvectors of the *i*^*-th*^ green space on the first and second principal components, respectively; $\epsilon_{ij}$ is the residual error term.

### Uncertainly analysis and Sensitivity analysis

Uncertainty analysis provides a critical basis for evaluating the reliability and robustness of carbon stock and estimates, ensuring that the findings can support informed decision-making [24]. In the context of life cycle carbon assessment, key sources of uncertainty include species-specific biomass equations, carbon content rates, root-to-shoot ratios, and emission factors associated with construction, irrigation, pruning, pesticide application, and end-of-life disposal. To assess the influence of these parameters on the final carbon estimates, a sensitivity analysis was conducted in which each parameter was varied within a plausible range based on measurement errors, field variability, or literature-reported values, while all other parameters were held constant. The resulting variations in life cycle carbon storage, carbon emissions, and net carbon balance were used to identify the parameters to which the outputs were most sensitive [25]. This approach allows quantification of the relative contributions of biological, management, and methodological uncertainties, and highlights the factors that most strongly affect the reliability of urban tree carbon assessments over their entire life cycle.

## Results

### Carbon storage of main tree species in different green space types

**Fig 1:**
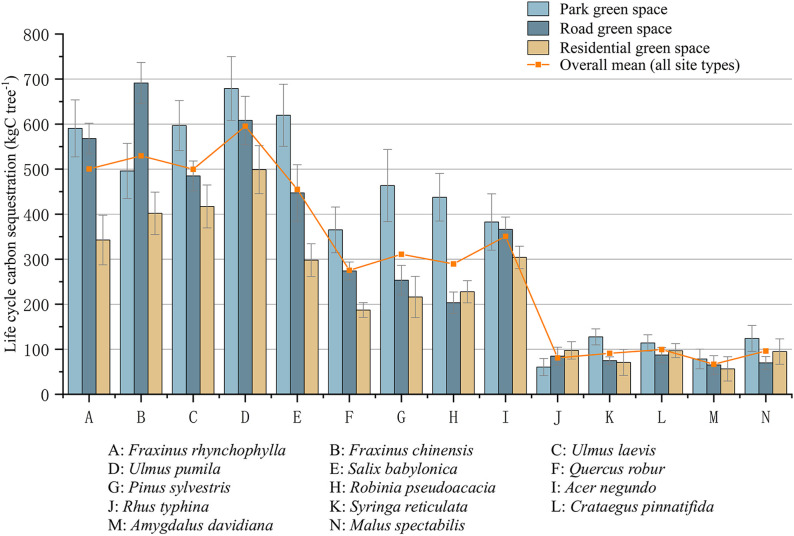
Life cycle carbon sequestration of major urban tree species across different green space types Error bars: mean ± SD, n = 3 sites × 10 trees/species.

Assessing life cycle carbon storage across different urban green space types is essential for evaluating the long-term carbon mitigation potential of urban vegetation in arid cities such as Shihezi. Using a life cycle assessment framework, the average per-tree carbon storage of 14 commonly planted tree species was quantified for park, roadside, and residential green spaces (Fig 1). The results revealed pronounced interspecific differences in life cycle carbon storage, as well as clear effects of green space type. Overall, large tree species exhibited substantially higher life cycle carbon storage than small tree species, reflecting their greater cumulative biomass accumulation over the defined urban application lifespan. Among all species, *Ulmus pumila*, *Fraxinus chinensis*, *Fraxinus rhynchophylla*, *Ulmus laevis*, and *Salix babylonica* showed the highest average life cycle carbon storage across the three green space types. In contrast, small ornamental species, including *Amygdalus davidiana*, *Rhus typhina*, and *Syringa reticulata*, consistently exhibited lower carbon storage values. Across green space types, park green spaces generally supported higher life cycle carbon storage per tree than roadside and residential green spaces for most species. For example, *Salix babylonica*, *Pinus sylvestris*, and *Robinia pseudoacacia* accumulated substantially more carbon over their life cycle in park environments than in roadside or residential settings. This pattern may be related to general differences in site conditions among green space types. Park green spaces typically provide less constrained growing environments, whereas roadside and residential green spaces are subject to stronger spatial and management limitations, which may restrict cumulative biomass accumulation over the application lifespan.

### Carbon emissions of main tree species in different green space types

The life cycle carbon emissions of 14 tree species investigated in park green space, street green space and residential green space are shown in Table 2. Among all species and green space types, carbon emissions in the maintenance phase were dominant, while planting and tree removal contributed less to total life-cycle emissions. Among them, the emissions related to maintenance and management ranged from 153.9 to 536 kgC tree^-1^. In contrast, tree planting carbon emissions were relatively stable in various green space types, generally between 5.4–18.9 kgC tree^-1^. Carbon emissions associated with tree disposal have been the smallest component of the life cycle, ranging from 2.8 to 15.6 kgC tree^-1^.

There are significant differences in the life cycle carbon emissions of different green space types. For most species, the total life cycle carbon emissions were the highest in park green space, the middle in roadside green space, and the lowest in residential green space, reflecting the differences in cumulative carbon emissions throughout the application period. For example, the life cycle carbon emission of *Ulmus pumila* in park green space is 540.1 kgC tree^-1^, while the maintenance emissions in roadside green space and residential green space are 506.1 kgC tree^-1^ and 337.6 kgC tree^-1^, respectively. In all green space types, the life cycle carbon emissions of small ornamental tree species are lower than those of large tree species.

**Fig 2:**
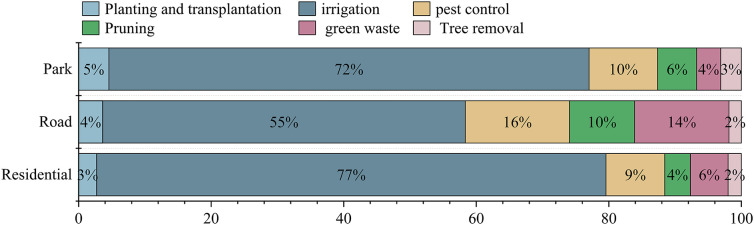
Relative contribution of different emissions factors to carbon emissions.

The contribution of maintenance activities to carbon emissions differed among green space types (Fig 2). Irrigation dominated emissions across all green spaces, accounting for 55–77% of the total, followed by pest control and pruning. Roadside green spaces showed relatively higher contributions from pest control and green waste management, whereas planting, transplantation, and tree removal consistently contributed less than 5% across all green space types (Table 2).

**Table 2:** Lifecycle carbon emissions (CE) of different tree species across urban green space types.

Species	Green space	Planting and transplantation/ kgC tree^-1^	Maintenance/kgC tree^-1^				Tree removal/ kgC tree^-1^	Total carbon Emission/ kgC tree^-1^
			irrigation/ kgC tree^-1^	Pesticide/kgC tree^-1^	Pruning/ kgC tree^-1^	green waste/ kgC tree^-1^		
*Fraxinus rhynchophylla*(A1）	Ⅰ	16.49	423.00	54.50	28.50	30.00	9.25	561.74
	Ⅱ	18.86	235.50	76.50	53.00	70.00	6.88	460.74
	Ⅲ	16.98	244.50	42.50	27.00	8.50	8.76	348.24
*Fraxinus chinensis*(A2)	Ⅰ	16.45	388.00	53.50	31.00	31.50	9.16	529.61
	Ⅱ	18.70	203.50	96.00	70.50	89.50	6.91	485.11
	Ⅲ	16.94	224.00	43.00	28.50	11.50	8.67	332.61
*Ulmus laevis*(A3)	Ⅰ	16.49	431.00	49.50	20.50	24.50	15.57	557.56
	Ⅱ	18.74	255.50	81.50	59.50	77.00	13.32	505.56
	Ⅲ	16.98	251.00	27.50	16.50	10.50	15.08	337.56
*Ulmus pumila*(A4)	Ⅰ	16.49	353.50	66.00	44.50	44.00	15.57	540.06
	Ⅱ	18.74	175.00	110.50	85.00	103.50	13.32	506.06
	Ⅲ	16.98	205.00	49.00	33.50	18.00	15.08	337.56
*Salix babylonica*(A5)	Ⅰ	16.49	410.50	48.00	27.50	26.50	15.30	544.29
	Ⅱ	18.74	226.50	86.50	61.00	79.50	13.05	485.29
	Ⅲ	16.98	239.00	43.50	27.50	12.50	14.81	354.29
*Quercus robur*(A6)	Ⅰ	16.58	419.00	55.00	28.50	31.50	9.38	559.96
	Ⅱ	18.83	283.00	61.50	41.50	51.50	7.13	463.46
	Ⅲ	17.07	242.50	39.50	28.50	3.50	8.89	339.96
*Pinus sylvestris*(A7)	Ⅰ	16.49	406.50	70.50	8.50	43.00	9.11	554.1
	Ⅱ	18.74	266.50	88.00	19.00	59.00	6.86	458.1
	Ⅲ	16.98	235.50	47.00	16.50	12.50	8.62	337.1
*Robinia pseudoacacia*(A8)	Ⅰ	16.49	449.50	29.50	13.00	28.50	15.57	552.56
	Ⅱ	18.74	293.00	54.00	31.50	52.00	13.32	462.56
	Ⅲ	16.98	273.50	21.00	10.50	17.00	15.08	354.06
*Acer negundo*(A9)	Ⅰ	16.45	426.50	39.00	16.00	22.50	9.04	529.49
	Ⅱ	18.70	311.00	57.00	43.00	45.00	6.79	481.49
	Ⅲ	16.94	249.50	27.00	15.00	16.50	8.55	333.49
*Rhus typhina*(B1)	Ⅰ	5.37	231.30	23.10	4.50	19.20	6.18	289.65
	Ⅱ	6.43	125.40	34.50	12.60	33.30	5.12	217.35
	Ⅲ	5.69	130.80	12.30	4.50	6.30	5.86	165.45
*Syringa reticulata*(B2)	Ⅰ	5.40	276.60	15.60	8.10	18.00	3.81	327.51
	Ⅱ	6.46	185.70	25.50	20.40	33.90	2.75	274.71
	Ⅲ	5.72	157.80	10.20	6.90	9.60	3.49	193.71
*Crataegus pinnatifida*(B3)	Ⅰ	5.40	236.10	21.60	9.60	16.20	3.81	292.71
	Ⅱ	6.46	133.80	30.30	13.20	35.10	2.75	221.61
	Ⅲ	5.72	140.70	15.30	4.20	6.30	3.49	175.71
*Amygdalus davidiana*(B4)	Ⅰ	5.40	239.40	21.60	2.40	17.70	3.81	290.31
	Ⅱ	6.46	141.90	34.20	13.50	33.00	2.75	231.81
	Ⅲ	5.72	136.50	15.30	7.50	5.10	3.49	173.61
*Malus spectabilis*(B5)	Ⅰ	5.40	271.20	25.20	7.50	13.50	3.81	326.61
	Ⅱ	6.46	193.20	34.20	20.10	26.10	2.75	282.81
	Ⅲ	5.72	147.00	17.40	7.20	6.90	3.49	187.71

Note:Ⅰ: park green space; Ⅱ: road green space; Ⅲ: residential green space.

### Carbon budget of main tree species in different green space types

**Fig 3:**
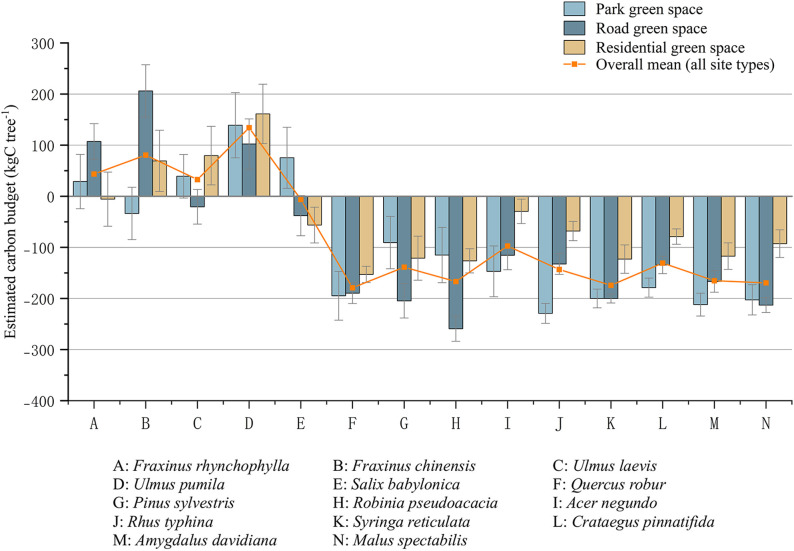
Life cycle carbon budget of major urban tree species across different green space types. Error bars: mean ± SD, n = 3 sites × 10 trees/species.

As shown in Fig 3, there are significant differences in the life cycle carbon budget of 14 tree species among different urban green space types. Among all species and green space types, the life cycle carbon budget ranged from −259.24 to 206.10 kgC tree^-1^. Obvious stratification of life cycle carbon budget was observed between large trees and small trees, and large trees usually had higher carbon budget than small trees. Under the defined life cycle boundary and current management system, *Ulmus pumila* showed the highest average life cycle carbon budget in the three green space types, indicating that there was a continuous net carbon sink in its life cycle. Other large tree species, including *Fraxinus rhynchophylla* and *Ulmus laevis*, showed positive carbon budgets only in some green space types. In all green space types, all small tree species showed negative life cycle carbon budget. It is worth noting that some large trees, including *Quercus robur*, *Pinus sylvestris* and *Robinia pseudoacacia*, showed negative life cycle carbon budgets throughout the life cycle despite their high biomass potential.

### Differences of plant carbon budget under different environmental conditions

The GGE biplot is an effective tool for identifying the best-performing genotype adapted to a specific environment, the most stable genotype across multiple environments, and even the most representative environment for a given genotype (cultivar ecoregion-ME). In the decomposition of genotype and G × E interaction effects for large and small tree species, the first two principal components (PCs) were considered. The biplots for large and small trees explained 95.61% and 88.18% of the total observed variation, respectively. For large trees, the first and second PCs accounted for 84.4% and 11.21% of the variation, whereas for small trees, they accounted for 73.22% and 14.96%, respectively.

**Fig 4:**
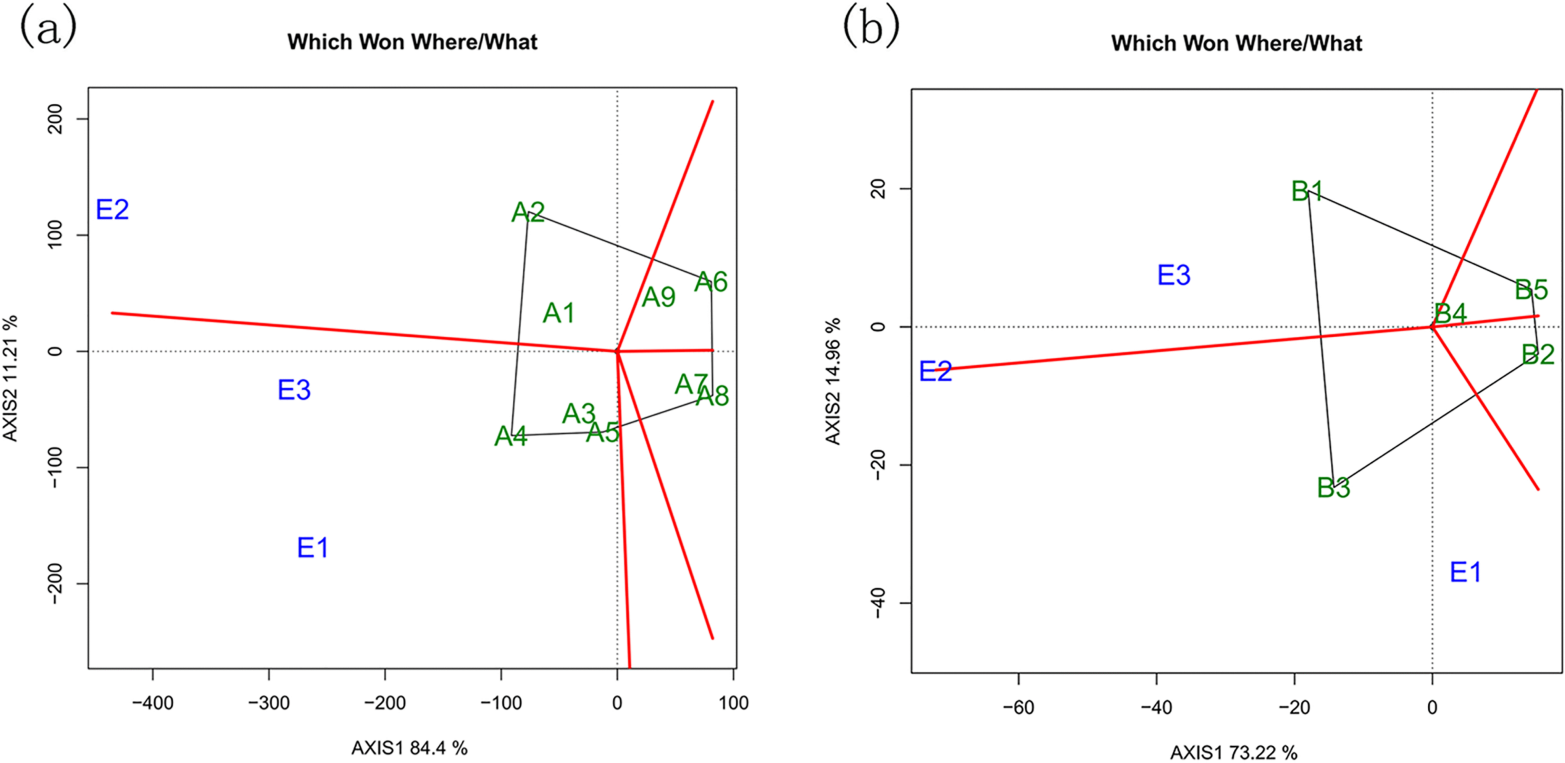
(a) represents the large tree species with the best carbon budget level in different green space environments and the green space environment group; (b) represents the small tree species with the best carbon budget level in different green space environments and green space environment grouping. The “which-won-where” pattern in the GGE biplot identifies which genotypes perform best under specific environmental conditions, illustrating species  ×  environment interactions across urban green space types.

The ‘which-won-where’ pattern highlights the genotypes that perform best in specific environments and provides a visual illustration of genotype × environment interactions across all tested sites. In the Fig 4, test environments are grouped based on G × E interactions among the top-performing genotypes. The genotype located at the vertex of each polygon performs best in the environments falling within its sector. In Fig 4a, the three environments were cut into 2 groups by the lines (red) that came out of the origin of the biplot. The A2 genotype had the best performance in E2; the A4 and A5 had the best performance in E1 and E3. The A6 and A8 genotype is present in a sector that does not contain environments. In Fig 4b, the three environments were cut into 2 groups, the groups are formed by E1 and E3. The B1 genotype had the best performance in E3; the B3 was considered with the best performance in E1, so these are the most adapted genotypes in these environments. The B2 and B5 genotype is present in a sector that does not contain environments, meaning that this genotype is the worst genotype in relation to carbon budget in some or all environments.

**Fig 5:**
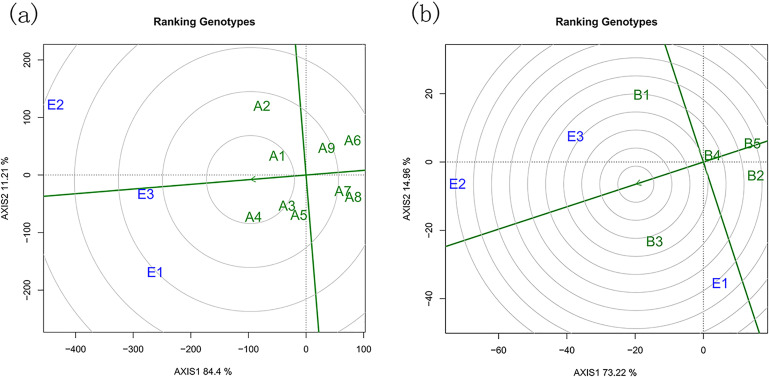
(a) and (b) respectively represent the comprehensive ranking of carbon budget of large trees and small trees in different green space environments.

The “environment ranking” model was used to evaluate plant stability. In this model, the vertical axis of the biplot represents the mean plant carbon budget level; plants located to the right of the origin have above-average values. The closer a plant is to the central concentric circle, the greater its stability. In Fig 5a, by comparing with the simulated ideal tree species and ranking based on the distance between the plotted points and the concentric circles, the stability ranking for large trees was as follows: *Fraxinus rhynchophylla* > *Ulmus pumila* > *Ulmus laevis* > *Salix babylonica* > *Fraxinus chinensis* > *Acer negundo* > *Pinus sylvestris* > *Robinia pseudoacacia* > *Quercus robur.* For small trees (Fig 5b), the stability ranking was: *Crataegus pinnatifida* > *Amygdalus davidiana* > *Rhus typhina*> *Syringa reticulata* > *Malus spectabilis*.

### The correlation between plant carbon storage, carbon emissions and influencing factors

**Fig 6:**
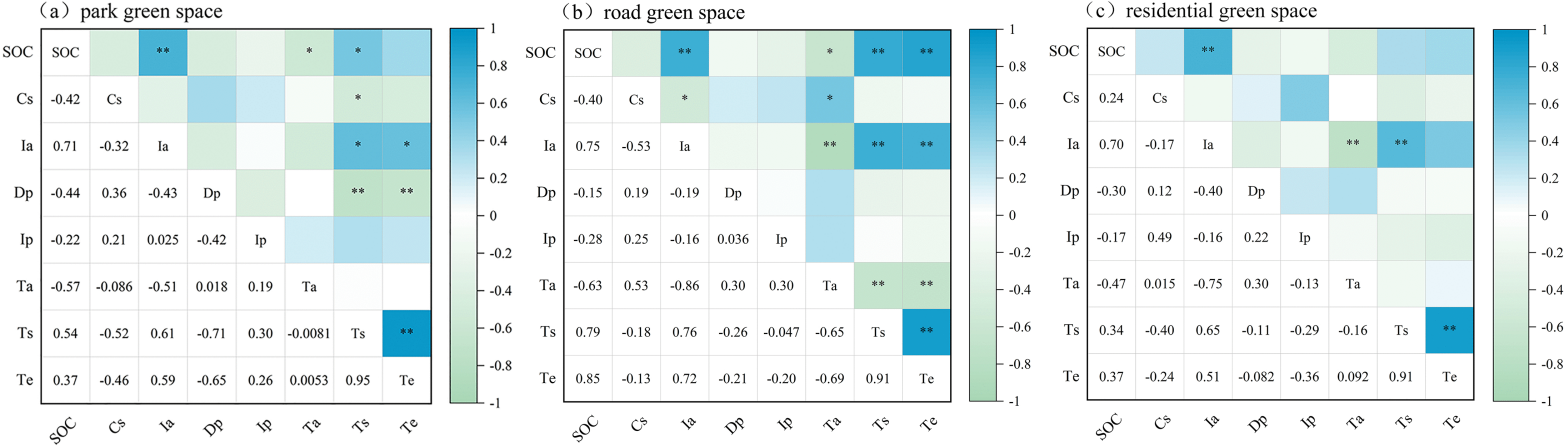
Correlation analysis of plant carbon storage, carbon emissions and influencing factors. Influencing factors refer to CS: soil compaction; SOC: soil organic carbon; Ia: Annual irrigation volume; Dp: density of planting; Ip: pruning intensity; Ta: air temperature; Ts: carbon storage; Te: carbon emissions. **P < 0.01; *P < 0.05.

Understanding the relationship between environmental management variables and the carbon budget can provide an indirect basis for tree species selection and optimization of management practices in urban green spaces in arid regions. Therefore, factors such as soil compaction (Cs), soil organic carbon (SOC), annual irrigation volume (Ia), planting density (Dp), pruning intensity (Ip), and air temperature (Ta) were selected for Pearson’s correlation analysis with vegetation carbon storage (Ts) and carbon emissions (Te) (Fig 6) [26]. The correlation analysis revealed varying degrees of association between the vegetation carbon budget and environmental factors. Across all three green space types, Ts and Te were consistently and strongly positively correlated (park: r = 0.95; roadside: r = 0.91; residential: r = 0.91). Planting density showed moderate to strong negative correlations with Ts and Te in park green spaces (Ts: r = −0.71; Te: r = −0.65) and weaker negative associations in roadside and residential green spaces.

SOC and annual irrigation amount were generally positively correlated with Ts and Te across green space types, with the strongest associations observed in roadside green spaces (SOC–Ts: r = 0.79; Ia–Ts: r = 0.76). Soil compaction exhibited negative correlations with Ts and Te in park and residential green spaces, whereas correlations were weaker in roadside green spaces. Air temperature showed weak or negligible correlations with Ts and Te in park green spaces but moderate negative correlations in roadside green spaces (Ts: r = −0.65; Te: r = −0.69),. The strength of the correlations between vegetation and environmental factors varied among green space types, with vegetation in roadside green spaces showing stronger associations with environmental factors than those in the other two types.

## Discussion

### Effects of tree species characteristics and management on carbon budget

This study systematically evaluated the carbon budgets of common urban tree species in an arid city using a life-cycle assessment approach, demonstrating that species traits and management intensity jointly determine whether urban trees function as net carbon sinks or net carbon sources. Previous studies have demonstrated that tree species composition, planting density, and soil characteristics significantly influence the carbon sequestration capacity of urban green spaces [27]. In the present study, large trees consistently accumulated more carbon than small trees, consistent with Wang et al. [28], who identified individual tree size as a primary determinant of woody plant carbon storage, with larger trees generally exhibiting greater sequestration potential. Nevertheless, while increased maintenance intensity can enhance plant growth and ecosystem services, it inevitably incurs additional energy and resource inputs, thereby contributing to higher carbon emissions.

Among the tree species analyzed, only *Ulmus pumila*, *Fraxinus rhynchophylla*, *Fraxinus chinensis* and *Ulmus laevis* showed positive life cycle carbon budget in the average of the three urban green space types, acting as carbon sink; the other tree species are shown as carbon sources. These findings echo the life-cycle assessment of *Platanus racemosa* in Los Angeles by McPherson et al., where semi-arid conditions combined with intensive maintenance led to net carbon emissions over a 50-year period, with irrigation identified as the predominant source—exceeding emissions from planting or removal [29]. In our study, maintenance-phase emissions similarly dominated, with irrigation contributing over half of the total life-cycle carbon costs for most species. Notably, water itself does not emit CO₂; associated emissions arise indirectly from energy and material inputs required for extraction, treatment, and delivery [30]. In Shihezi, reclaimed or tap water irrigation produced approximately 3.35 Mg ha^-1^ yr^-1^ in annual carbon emissions, contrasting sharply with 0.02 Mg ha^-1^ yr^-1^ reported in humid regions such as Shanghai [31]. Strohbach et al. [32] found that in Leipzig, irrigation is only carried out during severe droughts, and therefore is not included in daily emissions. Its carbon emissions are mainly from the use of mechanical equipment, so urban trees can achieve positive carbon sinks in a relatively short period of time (about 5 years) after planting. However, the suitability of low-frequency irrigation strategies is highly climate-dependent and may not be appropriate for arid cities such as Shihezi. Under conditions of persistent water deficit and high evapotranspiration demand, irrigation is essential for maintaining tree physiological functioning and the ecosystem services they provide [33]. Combining the selection of drought-tolerant species with precision irrigation based on species-specific water requirements can improve irrigation efficiency [34]. This optimization may substantially reduce the carbon emissions associated with urban water management and potentially shift many urban tree species from marginal carbon sinks to strong net carbon sinks, thereby maximizing the climate mitigation potential of urban forests in arid environments.

### Species-environment interactions and stability

The carbon sink capacity of trees varies greatly depending on species selection [35] and planting locations [36]. GGE biplot analysis revealed differences in performance and stability among tree species across various types of urban green spaces. Consequently, species such as *Fraxinus rhynchophylla* and *Ulmus pumila*, which combine high stability with moderate carbon balance, are likely suitable as priority species for urban greening under conditions of environmental uncertainty. When implementing restoration or greening interventions across different green space types, selecting species that are better adapted to local conditions can maximize the ecological benefits of urban vegetation [37]. This tight coupling of tree species and habitats means that a carbon sink tree species that performs well in arid areas may not necessarily show equal advantages in humid areas due to differences in competitiveness or management, and vice versa. Pearson correlation analysis further indicated that planting density, soil organic carbon content, irrigation volume, and soil compaction significantly influence both carbon storage and carbon emissions, with these effects being particularly pronounced in roadside green spaces. Air temperature impacts the carbon budget differently by green space type. In parks, the correlation is weak, while roadside areas show a moderate negative correlation with carbon storage and emissions. The weak temperature response in parks may reflect more stable microclimatic conditions, where canopy cooling and regular irrigation reduce temperature variability and mitigate heat-related water stress. In contrast, roadside environments are more exposed to heat accumulation from impervious surfaces and greater disturbances. Elevated temperatures in these areas may enhance respiratory carbon losses and weaken the carbon sink capacity of trees [38]. Therefore, optimizing plant structure, density, and height composition according to the type and function of green space is recommended to enhance ecological performance [39]. Future urban forestry planning should therefore consider the combined effects of management intensity, water source, and species selection. Local genotypes with high photosynthetic efficiency and drought tolerance should be prioritized to maintain productivity under warming and water-limited conditions [40]. Integrating such species with efficient irrigation and minimal mechanical maintenance will help enhance the resilience and carbon efficiency of urban green spaces.

### Uncertainty and robustness of life cycle carbon budget estimation

The localized tree species equation and emission factors established in this study inevitably introduce uncertainty [41,42]. As reflected by the model diagnosis (S2 Table), some species showed relatively high standard error of parameters, indicating that the variability of biomass prediction was large, which may be related to the typical high structural variability of urban trees. The canopy morphology and growth pattern were greatly affected by management measures and site conditions. Therefore, it is necessary to recognize the uncertainty caused by the heterogeneity of the method when directly comparing the carbon budget results of this study with other studies based on different equation systems.

The uncertainty of each tree species was quantified by Monte Carlo simulation (Table 3), incorporating ±3% and ±8% relative standard deviations for DBH and tree height, log-normal distributions for biomass equations (CV = 0.10–0.27), ± 20% triangular variation for root-to-shoot ratios, 10−20% SD for management-related emissions, and a uniform distribution of 0.47–0.53 for the biomass-to-carbon conversion factor. By transmitting model-form and parameter variability through the full calculation chain, this approach allows uncertainty in allometric relationships to be directly reflected in the resulting carbon storage, emissions, and net carbon budget estimates. The results confirmed that although there were fluctuations at the parameter level, the overall ranking trend of carbon performance among species remained stable. In LCA studies with high data heterogeneity, single-point valuation should be explained in combination with its confidence interval [43]. For example, although the carbon budget intervals of individual small trees overlap at a 95% confidence level, large trees consistently show significantly higher climate benefits, indicating that the core conclusions of the study are statistically robust. Sensitivity analysis (±50% for four key input parameters) [44] indicated that irrigation and pesticide use contributed most to total emissions (Table 4).

**Table 3:** Monte Carlo–derived uncertainty of carbon stock, carbon emissions, and carbon budget.

Species	Mean CS	95% CI	Mean CE	95% CI	Mean C	95% CI
Ulmus laevis	751.7	500.0–1083.9	556.2	387.1–726.0	195.5	−121.2– + 568.3
Pinus sylvestris	560.0	412.0–743.9	554.0	392.5–714.3	5.9	−217.3– + 248.8
Amygdalus davidiana	121.3	71.9–180.9	289.4	196.2–384.1	−168.1	−274.4– − 59.7
Fraxinus_rhynchophylla	745.9	492.0–1075.0	536.4	372.0–704.1	209.5	−98.5– + 578.0
Fraxinus_chinensis	615.8	474.9–781.3	504.3	353.8–657.0	111.5	−97.8– + 333.0
Ulmus_pumila	844.7	635.8–1097.7	508.4	368.3–648.0	336.3	+81.4– + 623.8
Salix_babylonica	785.4	494.2–1171.4	511.9	350.5–671.1	273.5	−65.0– + 689.3
Quercus_robur	456.0	341.1–592.4	532.9	369.3–697.2	−77.0	−280.9– + 138.1
Robinia_pseudoacacia	549.7	371.4–782.1	521.9	349.7–701.1	27.9	−230.8– + 318.8
Acer_negundo	477.9	352.2–628.2	501.9	331.1–668.9	−24.0	−236.1– + 201.3
Rhus_typhina	107.4	69.2–159.1	277.6	186.6–366.7	−170.2	−269.6–-65.7
Syringa_reticulata	185.1	126.6–263.0	319.0	209.5–425.9	−133.9	−259.8–-1.7
Crataegus_pinnatifida	88.3	66.4–115.9	283.9	190.9–376.0	−195.6	−292.5–-99.3
Malus_spectabilis	161.4	90.7–267.8	317.4	212.7–425.4	−156.0	−287.5–-11.7

**Table 4:** The sensitivity analysis result.

Factor	Consumption	Factor	Carbon emission (kgC tree^-1^)	Assumptions consumption	Assumption emission (kgC tree^-1^)	sensitivity
Irrigation	11.36	1.85	5.73	17.04	8.60	2.87
Pruning	1.03	2.03	0.57	1.55	0.86	0.29
Pesticide spraying	0.24	16.5	1.09	0.36	1.64	0.55
Green waste management	0.91	2.17	0.54	1.37	0.81	0.27

Note: The ‘Assumed Consumption’ columns represent ±50% variations from baseline emission values to assess sensitivity. Baseline values were derived from field surveys and maintenance records.

### Research limitations and future prospects

The current findings of this study are only valuable for urban green space allocation and climate management similar to the observation conditions in Shihezi, because differences in irrigation, soil properties, and microclimate can significantly affect the life cycle carbon budget at the species level [45]. Due to the diversity of nursery (seedling) sources and the difficulty of tracking energy and material inputs between multiple locations, the nursery production stage is not within the scope of this study, and may underestimate the total emissions to a certain extent, but the impact is expected to be smaller compared to the maintenance stage spanning decades [46]. Future research is expected to further reduce uncertainty and improve the reliability of tree-scale carbon assessment through more accurate measurement techniques and improved methods. In addition, the inclusion of soil carbon and understory vegetation will help to achieve a more comprehensive assessment of urban ecosystem carbon budget.

## Conclusions

(1) Species differences largely determine per-plant carbon sink efficiency. Over the full life cycle, *Ulmus pumila*, *Fraxinus chinensis*, and *Fraxinus rhynchophylla* exhibited higher carbon storage and strong long-term sink potential, whereas smaller species like *Amygdalus davidiana* and *Rhus typhina* showed lower net carbon sinks due to higher pruning frequency and maintenance-related emissions.
(2) Tree carbon budget is influenced by green space type through management intensity. Large species generally act as net sinks, whereas small or intensively managed species may exhibit net emissions due to maintenance-related carbon costs.
(3) GGE biplot analysis showed that both species and environmental context influence life-cycle carbon budget. Large species, such as *Ulmus laevis*, generally maintained high carbon sequestration across green space types, while other species were more environment-dependent. These findings can guide the selection of tree species to optimize carbon storage and stability in urban landscapes.
(4) Environmental management variables showed clear associations with full life-cycle tree carbon budgets in urban green spaces. Planting density and soil compaction were generally negatively associated with carbon storage and emissions, while soil organic carbon and irrigation tended to be positively associated, particularly in roadside green spaces. Air temperature showed weaker or moderate associations depending on the green space type.

## Supporting information

S1 Table Basic data for biomass equations. (CSV)

S2 Table Parameters and diagnostics of biomass equations. (CSV)

S1 File Xinjiang Landscape Greening Engineering Consumption Quota. (PDF)

## References

[1] Lind E, Prade T, Sjöman Deak J, Levinsson A, Sjöman H. How green is an urban tree? The impact of species selection in reducing the carbon footprint of park trees in Swedish cities. Front Sustain Cities. 2023;5. doi: 10.3389/frsc.2023.1182408

[2] Demuzere M, Orru K, Heidrich O, Olazabal E, Geneletti D, Orru H, et al. Mitigating and adapting to climate change: multi-functional and multi-scale assessment of green urban infrastructure. J Environ Manage. 2014;146:107–15. doi: 10.1016/j.jenvman.2014.07.025 25163601

[3] Zhou W, Wang J, Qian Y, Pickett STA, Li W, Han L. The rapid but “invisible” changes in urban greenspace: A comparative study of nine Chinese cities. Sci Total Environ. 2018;627:1572–84. doi: 10.1016/j.scitotenv.2018.01.335 30857118

[4] Kong F, Yin H, James P, Hutyra LR, He HS. Effects of spatial pattern of greenspace on urban cooling in a large metropolitan area of eastern China. Landscape and urban planning. 2014;128:35–47. doi: 10.1016/j.landurbplan.2014.04.018

[5] Livesley SJ, McPherson GM, Calfapietra C. The Urban Forest and Ecosystem Services: Impacts on Urban Water, Heat, and Pollution Cycles at the Tree, Street, and City Scale. J Environ Qual. 2016;45(1):119–24. doi: 10.2134/jeq2015.11.0567 26828167

[6] Robitu M, Musy M, Inard C, Groleau D. Modeling the influence of vegetation and water pond on urban microclimate. Solar Energy. 2006;80(4):435–47. doi: 10.1016/j.solener.2005.06.015

[7] Churkina G. Modeling the carbon cycle of urban systems. Ecological Modelling. 2008;216(2):107–13. doi: 10.1016/j.ecolmodel.2008.03.006

[8] Kong L, Shi Z, Chu LM. Carbon emission and sequestration of urban turfgrass systems in Hong Kong. Sci Total Environ. 2014;473–474:132–8. doi: 10.1016/j.scitotenv.2013.12.012 24365589

[9] Nowak DJ, Stevens JC, Sisinni SM, Luley CJ. Effects of Urban Tree Management and Species Selection on Atmospheric Carbon Dioxide. isa. 2002;28(3):113–22. doi: 10.48044/jauf.2002.017

[10] Huang H, Roland-Holst D, Wang C, Cai W. China’s income gap and inequality under clean energy transformation: A CGE model assessment. Journal of Cleaner Production. 2020;251:119626. doi: 10.1016/j.jclepro.2019.119626

[11] Tang FY. Carbon Footprint Research of Landscape Engineering Based on Life Cycle Analysis — Take the Unoccupied Space Landscape Engineering of Wuhan Optics Valley Road (Optics Valley Road One — Liufang Road Section) for Example. AMM. 2014;584–586:695–704. doi: 10.4028/www.scientific.net/amm.584-586.695

[12] Park H-M, Jo H-K. Ecological Design and Construction Strategies through Life Cycle Assessment of Carbon Budget for Urban Parks in Korea. Forests. 2021;12(10):1399. doi: 10.3390/f12101399

[13] Jo H-K, McPherson GE. Carbon Storage and Flux in Urban Residential Greenspace. Journal of Environmental Management. 1995;45(2):109–33. doi: 10.1006/jema.1995.0062

[14] Amer A, Franceschi E, Hjazin A, Shoqeir JH, Moser-Reischl A, Rahman MA, et al. Structure and Ecosystem Services of Three Common Urban Tree Species in an Arid Climate City. Forests. 2023;14(4):671. doi: 10.3390/f14040671

[15] Naorem A, Jayaraman S, Dang YP, Dalal RC, Sinha NK, Rao ChS, et al. Soil Constraints in an Arid Environment—Challenges, Prospects, and Implications. Agronomy. 2023;13(1):220. doi: 10.3390/agronomy13010220

[16] Nogueira EM, Fearnside PM, Nelson BW, França MB. Wood density in forests of Brazil’s ‘arc of deforestation’: Implications for biomass and flux of carbon from land-use change in Amazonia. Forest Ecology and Management. 2007;248(3):119–35. doi: 10.1016/j.foreco.2007.04.047

[17] IPCC. IPCC Guidelines for National Greenhouse Gas Inventories. Hayama, Japan. 2006. http://www.ipcc-nggip.iges.or.jp/public/2006gl/index.html

[18] Lázaro-Lobo A, Ruiz-Benito P, Cruz-Alonso V, Castro-Díez P. Quantifying carbon storage and sequestration by native and non-native forests under contrasting climate types. Glob Chang Biol. 2023;29(16):4530–42. doi: 10.1111/gcb.16810 37287121

[19] Kim J-Y, Jo H-K. Estimating Carbon Budget from Growth and Management of Urban Street Trees in South Korea. Sustainability. 2022;14(8):4439. doi: 10.3390/su14084439

[20] Cai B, Zhu S, Yu S, Dong H, Zhang C, Wang C. The interpretation of 2019 refinement to the 2006 IPCC guidelines for national greenhouse gas inventory. Environmental engineering. 2019;37(8):1–11. doi: 10.13205/j.hjgc.201908001

[21] Institute of Environmental Planning. China Products Carbon Footprint Factors Database 2022. https://lca.cityghg.com/. Accessed 2023 October 21.

[22] Lal R. Carbon emission from farm operations. Environ Int. 2004;30(7):981–90. doi: 10.1016/j.envint.2004.03.005 15196846

[23] Yan W, Wu H. Application of GGE biplot analysis to evaluate genotype (G), environment (E), and G× E interaction on Pinus radiata: A case study. New Zealand Journal of Forestry Science. 2008;38(1):132–42.

[24] Hong J, Shen GQ, Peng Y, Feng Y, Mao C. Uncertainty analysis for measuring greenhouse gas emissions in the building construction phase: a case study in China. Journal of Cleaner Production. 2016;129:183–95. doi: 10.1016/j.jclepro.2016.04.085

[25] De Marco I, Riemma S, Iannone R. Uncertainty of input parameters and sensitivity analysis in life cycle assessment: An Italian processed tomato product. Journal of Cleaner Production. 2018;177:315–25. doi: 10.1016/j.jclepro.2017.12.258

[26] Xu J, Jiao A, Deng M, Ling H. Changes in ecosystem carbon sequestration and influencing factors from a ’Past-Future’ perspective: A case study of the Tarim River. Ecological Indicators. 2024;169:112861. doi: 10.1016/j.ecolind.2024.112861

[27] ZHOU Jian 周健, XIAO Rongbo 肖荣波, ZHUANG Changwei 庄长伟, DENG Yirong 邓一荣. The carbon sink of urban forests and efficacy on offsetting energy carbon emissions from city in Guangzhou. 生态学报. 2013;33(18):5865–73. doi: 10.5846/stxb201305030913

[28] YW A, QC A, XL B. Promoting sustainable carbon sequestration of plants in urban greenspace by planting design: A case study in parks of Beijing. Urban Forestry & Urban Greening. 2021. doi: 10.1016/j.ufug.2021.127291

[29] McPherson EG, Kendall A, Albers S. Life cycle assessment of carbon dioxide for different arboricultural practices in Los Angeles, CA. Urban Forestry & Urban Greening. 2015;14(2):388–97. doi: 10.1016/j.ufug.2015.04.004

[30] Zou X, Li Y e, Li K, Cremades R, Gao Q, Wan Y. Greenhouse gas emissions from agricultural irrigation in China. Mitigation and Adaptation Strategies for Global Change. 2015;20(2):295–315. doi: 10.1007/s11027-013-9492-9

[31] Xiao X, Chen T, Zheng Z. Study on carbon emission of greening maintenance of parks in Shanghai. Journal of Shanghai Jiaotong University (Agricultural Science). 2013;31(01):67–71. doi: 10.3969/J.ISSN.1671-9964.2013.01.013

[32] Strohbach MW, Arnold E, Haase D. The carbon footprint of urban green space—A life cycle approach. Landscape and Urban Planning. 2012;104(2):220–9. doi: 10.1016/j.landurbplan.2011.10.013

[33] Broadbent AM, Coutts AM, Tapper NJ, Demuzere M. The cooling effect of irrigation on urban microclimate during heatwave conditions. Urban Climate. 2018;23:309–29. doi: 10.1016/j.uclim.2017.05.002

[34] Rosenberger L, Leandro J, Helmreich B. Providing sufficient water for urban trees with limited root space during drought: Modeling of irrigation scenarios in a temperate climate. Urban Forestry & Urban Greening. 2025;104:128670. doi: 10.1016/j.ufug.2025.128670

[35] Zhang H, Feng Z, Shen C, Li Y, Feng Z, Zeng W, et al. Relationship between the geographical environment and the forest carbon sink capacity in China based on an individual-tree growth-rate model. Ecological Indicators. 2022;138:108814. doi: 10.1016/j.ecolind.2022.108814

[36] Watt MS, Kimberley MO. Spatial comparisons of carbon sequestration for redwood and radiata pine within New Zealand. Forest Ecology and Management. 2022;513:120190. doi: 10.1016/j.foreco.2022.120190

[37] Costello L. Urban Trees and Water: An Overview of Studies on Irrigation Needs in the Western United States and a Discussion Regarding Future Research. AUF. 2013;39(3). doi: 10.48044/jauf.2013.018

[38] Yan G, Wang Q, Han S, Guo Z, Yu J, Wang W, et al. Beneficial effects of warming on temperate tree carbon storage depend on precipitation and mycorrhizal types. Sci Total Environ. 2022;819:153086. doi: 10.1016/j.scitotenv.2022.153086 35038543

[39] Lahoti S, Lahoti A, Joshi RK, Saito O. Vegetation Structure, Species Composition, and Carbon Sink Potential of Urban Green Spaces in Nagpur City, India. Land. 2020;9(4):107. doi: 10.3390/land9040107

[40] Nitschke CR, Nichols S, Allen K, Dobbs C, Livesley SJ, Baker PJ, et al. The influence of climate and drought on urban tree growth in southeast Australia and the implications for future growth under climate change. Landscape and Urban Planning. 2017;167:275–87. doi: 10.1016/j.landurbplan.2017.06.012

[41] Yang C, Yao T, Shiming D, Jiang W. Carbon emissions accounting and uncertainty analysis in campus settings: A case study of a university in Sichuan, China. PLoS One. 2025;20(4):e0321216. doi: 10.1371/journal.pone.0321216 40238836 PMC12002547

[42] Vorster AG, Evangelista PH, Stovall AEL, Ex S. Variability and uncertainty in forest biomass estimates from the tree to landscape scale: the role of allometric equations. Carbon Balance Manag. 2020;15(1):8. doi: 10.1186/s13021-020-00143-6 32410068 PMC7227279

[43] Adams N, Allacker K. Parameter sensitivity and data uncertainty assessment of the cradle-to-gate environmental impact of state-of-the-art passive daytime radiative cooling materials. Environ Sci Eur. 2025;37(1). doi: 10.1186/s12302-025-01093-x

[44] Meng W, Hu B, Sun N, Mo X, He M, Li H. An integrated full cost model based on extended exergy accounting toward sustainability assessment of industrial production processes. Clean Techn Environ Policy. 2019;21(10):1993–2004. doi: 10.1007/s10098-019-01767-0

[45] Kelly J, Kljun N, Cai Z, Doerr SH, D’Onofrio C, Holst T, et al. Wildfire impacts on the carbon budget of a managed Nordic boreal forest. Agricultural and Forest Meteorology. 2024;351:110016. doi: 10.1016/j.agrformet.2024.110016

[46] Zhang Y, Meng W, Yun H, Xu W, Hu B, He M, et al. Is urban green space a carbon sink or source? - A case study of China based on LCA method. Environmental Impact Assessment Review. 2022;94:106766. doi: 10.1016/j.eiar.2022.106766

