# Supplementary material for: Carbon budget of common urban tree species in an arid city based on life cycle assessment
Source: PLoS One. 2026 Apr 24;21(4):e0345213. doi: 10.1371/journal.pone.0345213 (PMC13108748; doi:10.1371/journal.pone.0345213)
Supplement: S1 File — (PDF) [file pone.0345213.s003.pdf]

شثجاك ئۇيۇفۇر ثاپتونوم وايونى

The Xinjiang Uygur Autonomous Region

باغ -ء ثورماق ژه كزكرنش فورالۇشارشڈ سرهىاڈ مشدارىئى دپسالهلىئژ ثورمسى

Consumption Quota of Landscape Greening Engineering

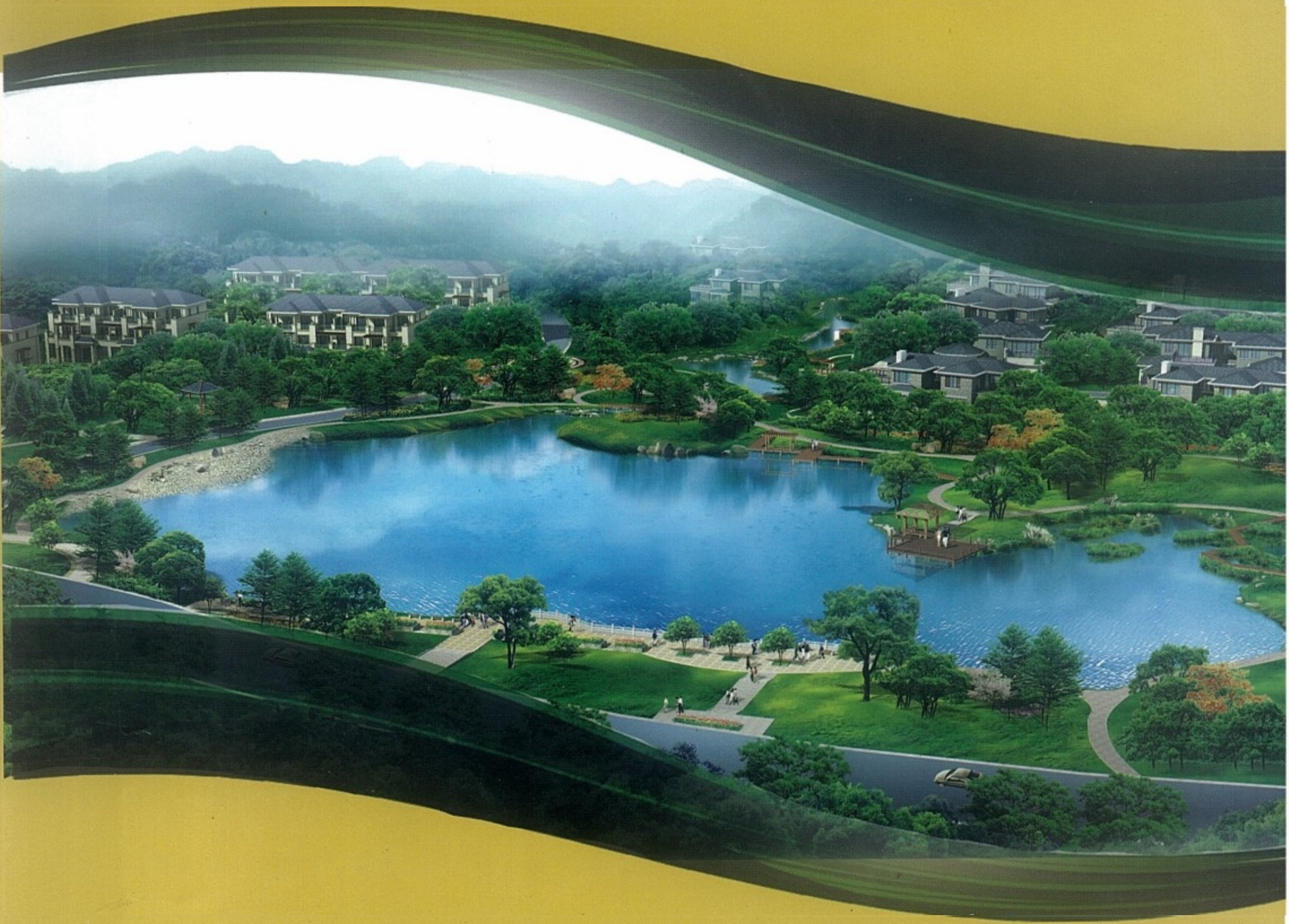

جۇككو قۇرۇلۇش ماتېرىيال سانائىت ناشرىياتى نهشر قىلدى

Zhongquan Building Materials Factory Food Service

CLIjuL

General STATEMENTS

1. The "Xinjiang Uygur Autonomous Region Landscape Greening Engineering Consumption Quota" (hereinafter referred to as this Quota) consists of five chapters: Chapter 1 on Greening Engineering, Chapter 2 on Garden Paths and Bridges, Chapter 3 on Landscape Engineering, Chapter 4 on General Items, and Chapter 5 on Measure Items, totaling 1,327 sub-items.

2. This quota is formulated based on the "National Unified Budget Quota for Ancient Architecture and Landscape Engineering in Urumqi Region" (1997), in compliance with national standards including the "Code for Quantity Pricing of Construction Projects" (GB50500—2013), "Code for Quantity Calculation of Landscape Engineering" (GB50858—2013), and the labor safety industry standard "Labor Quota for Construction Projects" (LD/T75.1~3—2008). It also incorporates current national product standards, design specifications, construction and acceptance standards, quality evaluation criteria, and safety technical operating procedures, all developed under normal construction conditions and rational organization, while considering the specific circumstances of our region.

3. This quota applies to new construction, expansion, and renovation of landscaping projects within the urban jurisdiction of the autonomous region, but excludes maintenance, temporary works, and landscaping upkeep projects.

4. This quota is a consumption standard designed to complement the national standards: "Code for Quantity Pricing of Construction Projects" (GB50858—2013) and "Code for Quantity Calculation of Landscape Gardening Projects" (GB50858—2013).

5. This quota serves as the unified standard for calculating the quantity of landscaping projects in our district, defining project categories and measurement units. It provides the basis for compiling regional unit price summaries, design estimates, and tender control prices. Additionally, it forms the foundation for developing construction investment estimation indicators and serves as a reference for bidding quotations, contract execution, and project settlements. Enterprises may also utilize it as a reference for quota formulation and internal management optimization.

VI. Regarding the quota consumption:

１、 Labor consumption: Not categorized by job type or technical level, expressed as comprehensive man-days. It includes basic labor, overtime labor, auxiliary labor, and labor margin difference.

２、 Material consumption: This includes primary materials, auxiliary materials, and miscellaneous materials, with corresponding losses accounted for. The scope covers transportation losses from the construction site warehouse, centralized storage locations, or on-site processing sites to the operation or installation sites, as well as construction operation losses and on-site storage losses.

3、 The consumption of machine shifts shall be determined based on the comprehensive allocation of normal and reasonable machinery.

4、 This quota covers both horizontal and vertical transportation of materials (equipment) from the construction site warehouse or designated storage point to the installation site.

7. The construction water and electricity quotas in this document are based on the actual availability of water and electricity at the site.

8. The main construction procedures are specified in this quota, while minor procedures, though not detailed, are all included within the quota.

9. This quota applies to construction projects in areas below 2000 meters above sea level.

10. In this quota, items marked as 'within ×××' or 'below ×××' include '××× itself'; those marked as 'outside ×××' or 'above ×××' exclude '××× itself'.

Catalogue

Chapter 1: Greening Work

Section 1: Green Space Development (050101)………………………………………………… (3)

explain……………………………………………………………………………………… (5)

rules for calculation of quantity of work……………………………………………………… (6)

1. clearing of trees……………………………………………………………………… (7)

2. Digging up tree roots………………………………………………………………… (7)

3. Excavation of shrubs and roots……………………………………………………… (8)

4. Remove the grass……………………………………………………………………… (8)

5. Roof cleaning………………………………………………………………………… (9)

6. returning of planting soil……………………………………………………………… (9)

7. Greening land consolidation………………………………………………………… (15)

8. Green slope modeling………………………………………………………………… (18)

9. Roof Garden Base Treatment………………………………………………………… (19)

Section 2: Planting Flowers and Trees (050102)……………………………………………… (21)

explain……………………………………………………………………………………… (23)

rules for calculation of quantity of work……………………………………………………… (24)

1. Planting trees………………………………………………………………………… (25)

2. big tree transplanting………………………………………………………………… (29)

3. planting shrubs……………………………………………………………………… (31)
4. planting bamboo…………………………………………………………………… (34)
5. Planting hedges…………………………………………………………………… (36)
6. climbing plant cultivation………………………………………………………… (37)
7. planting strip……………………………………………………………………… (37)
8. Planting flowers…………………………………………………………………… (38)
9. planting aquatic plants…………………………………………………………… (39)
10. Floral arrangement……………………………………………………………… (39)
11. Laying turf……………………………………………………………………… (40)
12. spray seeding…………………………………………………………………… (41)
13. planting grass in brick………………………………………………………… (42)
14. potting…………………………………………………………………………… (42)
15. Planting arrangement…………………………………………………………… (44)
Section 3: Sprinkler Irrigation in Green Spaces (050103)………………………… (45)
explain………………………………………………………………………………… (47)
rules for calculation of quantity of work…………………………………………… (48)
1. sprinkler pipeline………………………………………………………………… (49)
2. sprinkler fittings installation……………………………………………………… (52)
3. Composition and Installation of Water Meter…………………………………… (56)
4. Installation of sprinkler nozzles and quick-release water valves……………… (58)
5. Oiling of pipes and iron parts…………………………………………………… (61)
6. well body masonry………………………………………………………………… (64)
7. Installation of automatic intelligent sprinkler irrigation system……………… (64)
8. Fixed rate fertilization system installation……………………………………… (67)

emit

5. formwork for cast-in-place concrete flower stand column……………………………… (309)

6. formwork for cast-in-place concrete flower rack beam……………………………… (310)

7. formwork for cast-in-place concrete flower bed wall……………………………… (312)

8. cast-in-place concrete slab formwork……………………………… (313)

9. cast-in-place concrete table and stool formwork……………………………… (315)

10. Stone Bridge Arch Stone, Stone Arch Face Formwork……………………………… (316)

11. Other component templates……………………………… (317)

Section 3: Tree Support Framework, Grass Rope Wrapping Around Trunks, and Installation of Shade (Cold-Proof) Canopies (050403)……………………………… (319)

explain……………………………… (321)

rules for calculation of quantity of work……………………………… (322)

1. tree support……………………………… (323)

2. Rope wrapped around the tree trunk……………………………… (323)

3. Set up a shade (or cold) shelter……………………………… (324)

Section 4: Retaining Dams and Drainage Works (050404)……………………………… (325)

explain……………………………… (327)

rules for calculation of quantity of work……………………………… (328)

1. cofferdam……………………………… (329)

2. drain off water……………………………… (329)

Section 5 Off-site Transportation of Heavy Machinery……………………………… (331)

explain……………………………… (333)

rules for calculation of quantity of work……………………………… (334)

1. caterpillar excavating machine……………………………… (335)

2. crawler dozer……………………………… (335)

Chapter 1 Greening Project

Section 1: Green Space Development (050101)

Explain

1. This section includes 103 sub-items, such as felling trees, digging up tree roots (stumps), cutting and digging shrubs and roots, clearing turf, roof cleaning, planting soil backfilling (or replacement), landscaping land preparation, green space slope shaping, and roof garden base treatment.

2. All landscaping projects shall include earthwork transportation within 100 meters of the construction site. For distances exceeding 100 meters, the corresponding quota item shall apply for every 50 meters of excess (less than 50 meters shall be rounded up to 50 meters). The earthwork transportation quota item excludes disposal fees, which must be calculated separately.

3. The tree felling and root excavation project quota includes loading. If a platform lift truck is required for crown removal, the cost will be calculated separately.

4. The felling of trees, digging up tree roots (stumps), and cutting shrubs and their roots shall only be carried out on dead trees, and must be approved by the local administrative authorities.

5. The green slope design is suitable for terrain with a design elevation of up to 8cm and an average slope angle not exceeding 15°.

6. For all land used in landscaping projects, if the difference between the natural ground level and the designed ground level is within ±30 cm, the corresponding sub-item for manual landscaping land leveling shall be applied; if the difference exceeds ±30 cm, the respective quota sub-items for earth excavation or backfilling shall be applied.

7. Vertical transportation costs are excluded from the roof garden quota and shall be calculated separately when incurred.

Rules for Calculation of Quantity of Work

1. The quantity of trees felled and roots excavated shall be calculated by volume; the quantity of shrubs cut and excavated shall be calculated by volume; the area of turf mowed and ground cover plants removed shall be calculated by area.

2. The area of landscaping land shall be calculated based on the dimensions specified in the design drawings; the volume of screened soil shall be calculated based on the volume.

3. Demolition of various subgrade layers, foundation walls, and reinforced concrete shall be calculated by volume; pavement demolition by area; curb removal by length; surface water drainage by volume; and construction waste transportation by volume.

4. The slope design of green space shall be calculated based on the volume according to the dimensions specified in the design drawings.

5. Roof cleaning shall be calculated by area according to the dimensions shown in the design drawings; base treatment for rooftop gardens shall be calculated by area; installation of flexible drainage pipes shall be calculated by length; backfilling of filter layers with ceramic granules and planting soil shall be calculated by volume; and grout filling shall be calculated by length.

VI. Soil Replacement Project

1. The number of soil balls of trees was calculated according to the diameter of soil balls.

2. The number of bare-root trees is calculated according to different breast diameters, and the number of bare-root shrubs is calculated according to different heights.

3. The area of turf seeding is calculated according to different thicknesses.

4. The length of the hedge is calculated according to the height of the single and double rows.

5. The bamboo was calculated according to the number of different soil balls and breast diameter.

6. The number of climbing plants was calculated according to the diameter of the pit.

1. Clearing of Trees

Job description: Sawing down, pruning, cutting, digging, cleaning, and neatly stacking. Unit of measurement: plant

Quota number			1-1	1-2	1-3	1-4
Project			Tree felling, at a height of 20 cm above the ground, with the trunk diameter within 20 cm			
			30	40	50	Over 50
Name		Unit	Quantity			
Man-made	Combined work day	Work day	0. 304	0. 608	0. 903	2. 024

2. Digging up Tree Roots

Job description: Sawing down, pruning, cutting, digging, cleaning, and neatly stacking. Unit of measurement: plant

Quota number			1-5	1-6	1-7	1-8
Project			Remove the roots, and measure the trunk diameter at a height of 20 cm above the ground (within cm).			
			30	40	50	Over 50
Name		Unit	Quantity			
Man-made	Combined work day	Work day	0. 551	1. 093	1. 568	2. 185

3. Excavation of Shrubs and Roots

Job description: Shrub cutting, waste stacking, site cleaning. Unit of measurement: cluster

Quota number			1-9	1-10	1-11	1-12
Project			Excavate shrubs (within 1 cm of the crown)			
			100	150	200	250
Name		Unit	Quantity			
Man-made	Combined work day	Work day	0. 020	0. 040	0. 067	0. 115

4. Remove the Grass

Job duties: mowing grass, digging roots, clearing the site, and centrally stacking waste materials. Unit of measurement: $10m^2$

Quota number			1-13
Project			Remove the grass
Name		Unit	Quantity
Man-made	Combined work day	Work day	0. 204
Material	Other material costs	Yuan	0. 150

5. Roof Cleaning

Job responsibilities: Roof cleaning, debris removal, and on-site transportation. Unit of measurement: m^2

Quota number			1−14
Item			Roof cleaning
Name		Unit	Quantity
Man-made	Combined work day	Work day	0. 023
Machinery	Other machinery costs	Yuan	0. 080

6. Returning of Planting Soil

(1) Artificial soil replacement

① Joe, Shrub (with Ball of Soil)

Job description: Load and transport soil to the pit edge, etc. Unit of measurement: plant

Quota number			1−15	1−16	1−17	1−18	1−19
Project			The diameter of the soil ball in shrubs and trees is within (cm).				
			20	30	40	50	60
Name		Unit	Quantity				
Man-made	Combined work day	Work day	0. 010	0. 040	0. 050	0. 070	0. 140
Material	Planting soil	m^3	0. 020	0. 030	0. 080	0. 110	0. 210

Job description: Load and transport soil to the pit edge, etc. Unit of measurement: plant

Quota number			1-20	1-21	1-22	1-23	1-24
Project			The diameter of the soil ball in shrubs and trees is within (cm).				
			70	80	100	120	140
Name		Unit	Quantity				
Man-made	Combined work day	Work day	0. 190	0. 310	0. 430	0. 580	0. 770
Material	Planting soil	m³	0. 280	0. 500	0. 640	0. 870	1. 160

② naked tree

Job description: Load and transport soil to the pit edge, etc. **Unit of measurement: plant**

Quota number			1-25	1-26	1-27	1-28	1-29
Project			The diameter at breast height of the rootless tree is within (cm).				
			4	6	8	10	12
Name		Unit	Quantity				
Man-made	Combined work day	Work day	0. 020	0. 030	0. 060	0. 100	0. 150
Material	Planting soil	m³	0. 040	0. 080	0. 140	0. 250	0. 380

Job description: Load and transport soil to the pit edge, etc. Unit of measurement: plant

Quota number			1-30	1-31	1-32	1-33	1-34
Project			The diameter at breast height of the rootless tree is within (cm).				
			14	16	18	20	24
Name		Unit	Quantity				
Man-made	Combined work day	Work day	0. 230	0. 320	0. 430	0. 560	0. 810
Material	Planting soil	m³	0. 570	0. 800	1. 080	1. 410	2. 030

③ naked shrub

Job description: Load and transport soil to the pit edge, etc. **Unit of measurement: plant**

Quota number			1-35	1-36	1-37	1-38
Project			The height of the crown of bare-root shrubs is within (cm).			
			100	150	200	250
Name		Unit	Quantity			
Man-made	Combined work day	Work day	0. 010	0. 020	0. 030	0. 060
Material	Planting soil	m³	0. 020	0. 040	0. 080	0. 140

④ SEEDING AND GRASSING

Job description: Laying planting soil and rough plowing. Unit of measurement: 10m ²

Quota number			1-39	1-40	1-41
Project			Manual paving thickness (within cm)		
			30	40	50
Name		Unit	Quantity		
Man-made	Combined work day	Work day	0. 500	0. 600	0. 750
Material	Planting soil	m^3	3. 000	4. 000	5. 000

⑤ Hedgerow

Job description: Load and transport soil to the pit edge, etc. measurement unit： m

Quota number			1-42	1-43	1-44	1-45	1-46	1-47
Project			Hedge height (in meters)					
			0. 6	0. 8	1	1. 2	1. 5	2
Name		Unit	Quantity					
Man-made	Combined work day	Work day	0. 013	0. 017	0. 022	0. 026	0. 046	0. 066
Material	Planting soil	m^3	0. 075	0. 100	0. 125	0. 150	0. 240	0. 350
Machinery	Other machinery costs	Yuan	0. 005	0. 007	0. 008	0. 010	0. 030	0. 040

Job description: Load and transport soil to the pit edge, etc.

measurement unit：m

Quota number		1-48	1-49	1-50	1-51	1-52	1-53
Project		Height of double hedge (within m)					
		0.6	0.8	1	1.2	1.5	2
Name	Unit	Quantity					
Man-made: Combined work day	Work day	0.016	0.021	0.027	0.032	0.063	0.079
Material: Planting soil	m^3	0.160	0.213	0.299	0.320	0.400	0.600
Machinery: Other machinery costs	Yuan	0.010	0.013	0.017	0.020	0.040	0.040

⑥ Clump (scattered) Bamboo

Job description: Load and transport soil to the pit edge, etc.

Unit of measurement: 10 strains

Quota number		1-54	1-55	1-56
Project		Clump Bamboo Diameter (cm) × Depth (cm)		
		90×60	100×70	110×70
Name	Unit	Quantity		
Man-made: Combined work day	Work day	0.701	0.947	1.095
Material: Planting soil	m^3	3.030	4.090	4.730
Machinery: Other machinery costs	Yuan	0.390	0.530	0.620

Job description: Load and transport soil to the pit edge, etc. Unit of measurement: 10 strains

Quota number			1-57	1-58	1-59	1-60	1-61
Project			Dispersed bamboo diameter (within cm)				
			2	4	6	8	10
Name		Unit	Quantity				
Man-made	Combined work day	Work day	0. 154	0. 231	0. 344	0. 651	1. 035
Material	Planting soil	m^3	0. 664	0. 997	1. 488	2. 812	4. 472
Machinery	Other machinery costs	Yuan	0. 085	0. 128	0. 191	0. 362	0. 576

⑦ Climbing Plant Soil Replacement

Job description: Load and transport soil to the pit edge, etc. Unit of measurement: 100 units

Quota number			1-62	1-63	1-64	1-65
Project			Climbing plants (pit diameter cm × depth cm)			
			20×20	30×30	40×40	50×40
Name		Unit	Quantity			
Man-made	Combined work day	Work day	0. 145	0. 490	1. 163	1. 818
Material	Planting soil	m^3	0. 628	2. 120	5. 026	7. 854
Machinery	Other machinery costs	Yuan	0. 080	0. 280	0. 650	1. 020

(2) Mechanical Soil Replacement

Job description: Mechanical earth excavation, manual paving, and rough land leveling.　　Unit of measurement: $10m^2$

Quota number			1-66	1-67	1-68
Project			Mechanical soil replacement (thickness within cm)		
			30	40	50
Name		Unit	Quantity		
Man-made	Combined work day	Work day	0. 150	0. 200	0. 300
Material	Planting soil	m^3	3. 000	4. 000	5. 000
Machinery	Trailer shovel $8m^3$	Machine-team	0. 020	0. 030	0. 040

7. Greening Land Consolidation

(1) Artificially Cultivated Land for Afforestation

Job description: Clear the site, dig, fill and level within ±30cm, tidy up the green space, transport earth within 100 m, screen and so on.　　Unit of measurement:See table

Quota number			1-69	1-70
Project			Greening land consolidation	Sieve through native soil
			m^2	m^3
Name		Unit	Quantity	
Man-made	Combined work day	Work day	0. 050	0. 223

(2) Excavation of various road surfaces and subgrade layers

Job description: Excavation of lime-soil and gravel, demolition of concrete subgrade, pavement, curb stones, and centralized disposal of construction waste.

Unit of measurement: see table

Quota number			1-71	1-72	1-73	1-74
Project			Excavation of lime-soil sand and gravel concrete bedding	Excavation of Asphalt Concrete Slag Pavement and Subgrade	Demolition of all material pavements (excluding sub-grade)	Remove the curb stones.
			m^3		m^2	m
Name		Unit	Quantity			
Man-made	Combined work day	Work day	0.923	1.457	0.183	0.036

Job description: Digging ash soil, sand and gravel, concrete bedding, pavement, foundation wall, reinforced concrete, curb, slag soil medium. Unit of measurement:m^3

Quota number			1-75	1-76
Project			Excavation of foundation wall	Manual removal of rubble
Name		Unit	Quantity	
Man-made	Combined work day	Work day	1.160	0.702

(3) Surface Water Exclusion

Job description: Clear waterlogging and keep the surface clean after rain. Unit of measurement: m^3

Quota number			1-77
Project			Surface water exclusion
Name		Unit	Quantity
Man-made	Combined work day	Work day	0.040
Machinery	The outlet diameter of the single-stage centrifugal water pump is 100mm	Machine-team	0.022

(4) Mechanical Spoil

Job duties: soil loading, transportation, unloading, and road watering within the site. Unit of measurement: $10m^3$

Quota number			1-78	1-79	1-80	1-81
Project			Artificial soil filling	Mud and debris removal (within Km)		
				1	5	10
Name		Unit	Quantity			
Man-made	Combined work day	Work day	1.650	-	-	-
Material	Water	m^3	-	0.120	0.120	0.120
Machinery	Water truck with a capacity of 4000 liters	Machine-team	-	0.006	0.006	0.006
	8-ton dump truck	Machine-team	-	0.115	0.250	0.370

Job duties: soil loading, transportation, unloading, and road watering within the site. Unit of measurement: 10m 3

Quota number			1-82	1-83	1-84	1-85	1-86
Project			Mud and debris removal (within Km)				Shredded waste transportation (over 30 km)
			15	20	25	30	
Name		Unit	Quantity				
Material	Water	m³	0. 120	0. 120	0. 120	0. 120	0. 120
Machinery	Water truck with a capacity of 4000 liters	Machine-team	0. 006	0. 006	0. 006	0. 006	0. 006
	8-ton dump truck	Machine-team	0. 533	0. 695	0. 833	1. 042	1. 260

8. Green Slope Modeling

Job responsibilities: Conducting layout, installing elevation stakes, earthwork transportation within 50 meters, soil stacking, compaction, and slope shaping.

Unit of measurement: m ³

Quota number			1-87	1-88
Project			Green slope modeling	
			Machinery	Man-made
Name		Unit	Quantity	
Man-made	Combined work day	Work day	0. 016	0. 160
Material	Planting soil	m³	1. 100	1. 100
	Other material costs	Yuan	0. 040	–
Machinery	75kW tracked bulldozer	Machine-team	0. 006	–

9. Roof Garden Base Treatment

Job responsibilities: Site preparation, mortar mixing and transportation, plastering and curing.

Unit of measurement: $10m^2$

Quota number			1-89	1-90	1-91	1-92
Project			Waterproof mortar		Five-layer cement mortar construction method	
			Plane	Facade	Plane	Facade
Name		Unit	Quantity			
Man-made	Combined work day	Work day	1. 030	1. 540	1. 900	2. 450
Material	Cement mortar 1:2	m^3	0. 202	0. 211	0. 162	0. 163
	Plain cement slurry	m^3	–	-	0. 061	0. 061
	Water-proofing powder	kg	5. 560	5. 790	-	-
	Water	m^3	0. 380	0. 380	0. 380	0. 380
Machinery	200L mortar mixer	Machine-team	0. 055	0. 083	0. 102	0. 131

Job responsibilities: Leveling of filter layer and backfilling materials; laying geotextiles, stitching, and anchoring geotextiles.

Unit of measurement: see table

Quota number			1-93	1-94	1-95
Project			Graded gravel backfill for filter layer	Backfilling of filter layer with ceramsite	Geotextile filter layer
			m^2	m^3	m^2
Name		Unit	Quantity		
Man-made	Combined work day	Work day	0. 520	0. 090	0. 040
Material	Scree	Kg	1640. 000	–	–
	Haycite	m^3	–	1. 000	–
	Geotextile 250g	m^2	–	–	1. 200

Job description: Flexible water pipe: connection with branch pipes, cement fixation, etc.

measurement unit：m

Quota number			1-96	1-97	1-98	1-99	1-100
Project			Installation of flexible water pipe				
			Φ50	①80	Φ100	①150	Φ200
Name		Unit	Quantity				
Man-made	Combined work day	Work day	0. 050	0. 070	0. 090	0. 110	0. 130
Material	Cement mortar 1:2.5	m^3	0. 001	0. 001	0. 001	0. 001	0. 001
	Soft water-permeable pipe Φ50	m	1. 100	–	-	-	-
	Soft water-permeable pipe Φ80	m	-	1. 100	-	-	-
	Soft water-permeable pipe Φ100	m	-	-	1. 100	-	-
	Soft water pipe, Φ150	m	-	-	-	1. 100	–
	Soft water-permeable pipe Φ200	m	-	–	–	–	1. 100

Job Responsibilities: 1. Laying plastic barriers, suturing, and anchoring soil barriers. 2. Backfilling planting soil: leveling and preparing. 3. Construction

The oil paste was poured into the joint.

Unit of measurement: see table

Quota number			1-101	1-102	1-103
Project			Plastic grating	Backfilling planting soil	Building oil paste grouting
			m^2	m^3	m
Name		Unit	Quantity		
Man-made	Combined work day	Work day	0. 010	0. 280	0. 073
Material	Planting soil	m	-	1. 000	-
	Plastic grating	m^2	1. 200	-	-
	Construction grease	kg	-	-	1. 035
	Building varnish	kg	-	-	0. 068
	Other material costs	Yuan	-	-	0. 040

ژۇۋۇرۇنسۇئ ىنراھابىئ - لۇگ ۋۇ خرد - لاھ : ىفارئىراپ چنىكىئ

Section 2 Planting Flowers and Trees (050102)

Explain

1. This section includes 170 subcategories, such as planting trees, transplanting large trees, planting shrubs, planting bamboo, planting hedges, planting climbing plants, planting color bands, planting flowers, planting aquatic plants, three-dimensional flower arrangement, laying turf, spraying and planting grass (shrub) seeds, planting grass bricks, box/pot planting, and flower pot arrangement.

2. This section covers all construction phases including excavation (pit/slot), pruning, on-site tree transportation, box dismantling, planting, post installation, and watering maintenance.

3. The planting project in this section does not include the value of seedlings themselves. Seedlings shall be procured according to the species, specifications, and quantities specified in the design drawings, with corresponding loss rates calculated.

1. The loss rate of bare-rooted trees and shrubs is 1.5%.

2. The loss rate of hedge, color band and climbing plants was 2%;

3. the loss rate of bamboo, grass root, flower, grass seed and flower seed is 4%;

4. When the total height of flower beds is over 5m, the loss rate of finished card-pot flowers increases by 1% for every additional meter in height.

4. This section's three-dimensional floral modeling is not applicable to oversized or undersized individual pieces (1.5m or less, 10m or more).

5. All seedlings used in the planting project shall be procured and planted by the construction unit, with a survival rate of at least 95%. If the construction unit procures and supplies the seedlings independently, the survival rate shall be mutually agreed upon by both parties.

6. For excavation or planting of flowers and trees on slopes (slope>30°), the total man-days shall be calculated by multiplying the corresponding quota sub-item by the coefficient.

1.2 This regulation does not apply to sprinkler seeding for grass establishment.

7. The planting of aquatic plants is based on the assumption of no water. If planting in water is required, the cost will be calculated separately according to the actual conditions.

8. Where the construction unit cannot provide water supply at the landscaping project site, the costs shall be calculated separately according to the corresponding quota items for each type of planting.

9. If planted on a rooftop garden, additional vertical transportation costs shall be calculated.

10. This section excludes the erection and dismantling of construction scaffolding and mobile scaffolding. If such work is required, the associated costs shall be calculated separately.

XI. Regulations on Seedling Measurement

1. Trunk diameter: the diameter of the tree trunk measured at a height of 1.2 meters from the ground.

2. Diameter at ground level (DGL): The diameter of a tree trunk measured 10 cm above the ground surface.

3. Height of a tree: refers to the distance from the top of the tree to the ground surface.

4. Hedge height: refers to the vertical distance from the top of a green hedge plant to the ground surface.

5. Height of color strip: refers to the distance from the top of the color strip seedling to the ground surface.

6. Growth period: refers to the growth period from the planting of seedlings to the time of transplanting.

Rules for Calculation of Quantity of Work

1. Seedlings shall be calculated by unit (plant), cluster, meter, or square meter according to the type and specifications specified in the design drawings.

II. Seedling Planting:

1. The number of trees (shrub) with soil balls is calculated based on the diameter of the soil balls; the number of bare-root trees is calculated based on the different breast diameters; the number of bare-root shrubs is calculated based on the different crown heights.

2. Bamboo is divided into scattered bamboo and clustered bamboo. The number of scattered bamboo is calculated according to the different diameter at breast height (3~5 roots per plant), and the number of clustered bamboo is calculated according to the diameter of the root cluster(3~5 roots per cluster).

3. The length of the green hedge is calculated according to the different height of the single or double rows.

4. Climbing plants are counted by quantity according to their different growth years; wetland plants and emergent plants are counted by the number of nodes as shown in the design drawings; floating-leaved plants are counted by the number as shown in the design drawings.

5. Lawn, grass pavers, grass planting, color bands, and flowers shall be calculated based on the designed dimensions and green space projection area (this quota applies 25 perennial flowers/m^2,16 color bands/m^2,9 woody flowers/m^2, and 40 seasonal flowers/m^2). If the quantity per square meter differs from this quota, the number of seedlings may be adjusted according to design requirements. For spray-sown grass, the calculation shall be based on the designed dimensions and green space area according to different slope ratios and lengths. The placement of flower pots shall be calculated by quantity.

6. The arrangement of flowers is calculated according to the design requirements and the area of the design diagram.

7. The quantity of seedlings in wooden boxes is calculated according to different box specifications.

1. Planting Trees

(1) uprooting of trees (with soil ball)

Job description: Excavate pits, transport materials, and backfill with soil. Unit of measurement: plant

Quota number			1-104	1-105	1-106	1-107	1-108
Project			Excavation of trees (with soil balls) with soil ball diameter within (cm)				
			20	30	40	50	60
Name		Unit	Quantity				
Man-made	Combined work day	Work day	0. 036	0. 064	0. 118	0. 182	0. 309
Material	Straw rope	kg	1. 000	1. 500	2. 000	3. 000	4. 000

Note: When excavating exceptionally large or valuable trees, the cost shall be calculated separately.

Job description: Excavate pits, transport materials, and backfill with soil. Unit of measurement: plant

Quota number			1-109	1-110	1-111
Project			Excavation of trees (with soil balls) with soil ball diameter within (cm)		
			70	80	100
Name		Unit	Quantity		
Man-made	Combined work day	Work day	0. 382	0. 610	1. 320
Material	Straw rope	kg	4. 000	6. 000	10. 000
Machinery	5-ton truck-mounted crane	Machine-team	0. 011	0. 017	0. 020
	4-ton truck	Machine-team	0. 020	0. 030	0. 034

Note: When excavating exceptionally large or valuable trees, the cost shall be calculated separately.

(2) Planting of trees (with soil balls)

Work **content: digging holes, planting(falling holes, supporting, returning soil, compacting, building water enclosure), watering, covering soil, keeping moisture, shaping, cleaning.** Unit of measurement: plant

Quota number			1-112	1-113	1-114	1-115	1-116
Project			Planting of trees (with soil balls) with soil ball diameter within (cm)				
			20	30	40	50	60
Name		Unit	Quantity				
Man-made	Combined work day	Work day	0. 046	0. 082	0. 137	0. 209	0. 364
Material	Water	m^3	0. 025	0. 025	0. 050	0. 075	0. 100

Note: Extra calculation is required for planting exceptionally large or valuable trees.

Job responsibilities: Digging holes, planting (including hole placement, straightening, backfilling, tamping, and constructing water barriers), watering, covering with soil, _moisture retention, shaping, and cleaning. Unit of measurement: plant

Quota number			1-117	1-118	1-119
Project			Planting of trees (with soil balls) with soil ball diameter within (cm)		
			70	80	100
Name		Unit	Quantity		
Man-made	Combined work day	Work day	0. 391	0. 601	0. 965
Material	Water	m^3	0. 125	0. 150	0. 300
Machinery	5-ton truck-mounted crane	Machine-team	0. 011	0. 017	0. 020
	4-ton truck	Machine-team	0. 020	0. 030	0. 034

Note: Extra calculation is required for planting exceptionally large or valuable trees.

(3) uprooting of trees (naked roots)

Job description: Excavation, pit removal, pruning, slurry application, centralized transportation, and backfilling of the pit. Unit of measurement:plant

Quota number			1-120	1-121	1-122	1-123	1-124
Project			Excavated tree (bare root) with diameter at breast height (cm)				
			4	6	8	10	12
Name		Unit	Quantity				
Man-made	Combined work day	Work day	0. 036	0. 073	0. 118	0. 228	0. 364

Note: When excavating exceptionally large or valuable trees, the cost shall be calculated separately.

Job description: Excavation, pit removal, pruning, slurry application, centralized transportation, and backfilling of the pit. Unit of measurement: plant

Quota number			1-125	1-126	1-127
Project			Excavated tree (bare root) with diameter at breast height (cm)		
			14	16	18
Name		Unit	Quantity		
Man-made	Combined work day	Work day	0. 610	0. 692	0. 783
Machinery	5-ton truck-mounted crane	Machine-team	-	0. 008	0. 012
	4-ton truck	Machine-team	-	0. 014	0. 020

Note: When excavating exceptionally large or valuable trees, the cost shall be calculated separately.

(4) **Planting of trees (naked roots)**

Job responsibilities: Digging holes, planting (excavating holes, straightening, backfilling, tamping, constructing water barriers), watering, covering with soil, moisture retention, and shaping、put in order 。

Unit of measurement: plant

Quota number			1-128	1-129	1-130	1-131	1-132
Project			Planting of trees (bare-root) with trunk diameter within (cm)				
			4	6	8	10	12
Name		Unit	Quantity				
Man-made	Combined work day	Work day	0. 046	0. 082	0. 146	0. 246	0. 428
Material	Water	m³	0. 025	0. 050	0. 075	0. 100	0. 150

Note: When planting exceptionally large or rare trees, the cost shall be calculated separately.

Job responsibilities: Digging holes, planting (excavating holes, straightening, backfilling, tamping, constructing water barriers), watering, coveringwith soil, moisture retention, and shaping、put in order 。

Unit of measurement: plant

Quota Number			1-133	1-134	1-135
Project			Planting of trees (bare-root) with trunk diameter within (cm)		
			14	16	18
Name		Unit	Quantity		
Man-made	Combined work day	Work day	0. 692	0. 746	1. 010
Material	Water	m³	0. 200	0. 300	0. 400
Machinery	5-ton truck-mounted crane	Machine-team	-	0. 008	0. 012
	4-ton truck	Machine-team	-	0. 014	0. 020

Note: Extra calculation is required for planting exceptionally large or valuable trees.

2. Big Tree Transplanting

(1) Remove the tree (with its soil ball)

Job responsibilities: excavation, pruning, soil ball wrapping, tree trunk shaping, loading from the pit, and backfilling with soil. Measurement unit: per plant

Quota number			1−136	1−137	1−138	1−139	1−140	1−141	1−142
Project			Remove the tree (with its soil ball), where the soil ball diameter is within (cm).						
			120	140	160	180	200	240	280
Name		Unit	Quantity						
Man-made	Combined work day	Work day	1. 993	2. 493	4. 499	5. 787	7. 465	10. 664	13. 575
Material	Straw rope	kg	15. 000	20. 000	26. 310	33. 060	51. 750	74. 560	112. 000
Machin-ery	16-ton truck-mounted crane	Machine-team	0. 028	0. 040	0. 058	0. 083	0. 100	0. 150	0. 500

Note: When excavating exceptionally large or valuable trees, the cost shall be calculated separately.

(2) Planting a large tree (with a ball of soil)

Job responsibilities: Digging holes, planting (excavating holes, straightening, backfilling, tamping, constructing water barriers), watering, covering with soil, moisture retention, and shaping、put in order 。

Unit of measurement: plant

Quota number			1−143	1−144	1−145	1−146	1−147	1−148	1−149
Project			Planting large trees (with soil balls) with soil ball diameter within (cm)						
			120	140	160	180	200	240	280
Name		Unit	Quantity						
Man-made	Combined work day	Work day	1. 411	1. 818	2. 120	2. 695	4. 144	5. 578	8. 224
Material	Water	m^3	0. 400	0. 500	0. 700	1. 000	1. 400	1. 800	2. 500
Machin-ery	16-ton truck-mounted crane	Machine-team	0. 028	0. 040	0. 058	0. 083	0. 100	0. 150	0. 500

Note: When planting exceptionally large or rare trees, the cost shall be calculated separately.

(3) Remove the tree (bare roots)

Job responsibilities: excavation, pruning, soil ball wrapping, tree trunk shaping, loading from the pit, and backfilling with soil. Measurement unit: per plant

Quota number			1-150	1-151	1-152	1-153	1-154	1-155
Project			Remove large trees (with bare root diameter within cm)					
			20	25	30	35	40	45
Name		Unit	Quantity					
Man-made	Combined work day	Work day	1.056	1.537	2.018	3.142	4.368	6.818
Machinery	16-ton truck-mounted crane	Machine-team	0.020	0.028	0.040	0.056	0.072	0.100

Note: When excavating exceptionally large or valuable trees, the cost shall be calculated separately.

(4) Planting large trees (naked roots)

Work **content: digging holes, planting(falling holes, supporting, returning soil, compacting, building water enclosure), watering, covering soil, keeping moisture, shaping, cleaning**. Unit of measurement:plant

Quota number			1-156	1-157	1-158	1-159	1-160	1-161
Project			Planting large trees (with bare-root diameter within cm)					
			20	25	30	35	40	45
Name		Unit	Quantity					
Man-made	Combined work day	Work day	1.483	1.788	2.093	2.357	3.467	4.966
Material	Water	m^3	0.500	0.750	1.000	1.400	1.800	2.500
Machin-ery	16-ton truck-mounted crane	Machine-team	0.020	0.028	0.040	0.056	0.072	0.100

Note: When planting exceptionally large or rare trees, the cost shall be calculated separately.

3. Planting Shrubs

(1) Remove shrubs (with soil balls)

Job description: Excavation, soil ball wrapping, pit removal, centralized transportation, and backfilling. Unit of measurement: plant

Quota number			1−162	1−163	1−164	1−165	1−166
Project			Remove the shrub (with its soil ball), the diameter of which is within (cm).				
			20	30	40	50	60
Name		Unit	Quantity				
Man-made	Combined work day	Work day	0. 036	0. 073	0. 137	0. 209	0. 337
Material	Straw rope	kg	0. 500	1. 000	1. 500	2. 000	3. 000

Note: When excavating exceptionally large or valuable trees, the cost shall be calculated separately.

Job description: Excavation, soil ball wrapping, pit removal, centralized transportation, and backfilling. Unit of measurement: plant

Quota number			1−167	1−168	1−169	1−170	1−171
Project			Remove the shrub (with its soil ball), the diameter of which is within (cm).				
			70	80	100	120	140
Name		Unit	Quantity				
Man-made	Combined work day	Work day	0. 437	0. 692	1. 420	2. 129	2. 694
Material	Straw rope	kg	4. 000	6. 000	10. 000	15. 000	20. 000
Machinery	5-ton truck-mounted crane	Machine-team	0. 011	0. 017	0. 020	0. 028	0. 040
	4-ton truck	Machine-team	0. 020	0. 030	0. 034	0. 047	0. 068

Note: When excavating exceptionally large or valuable trees, the cost shall be calculated separately.

(2) Planting shrubs (with soil balls)

Work content : Digging, cutting (falling pit, righting, returning soil, tamping, building water surround), watering, covering, soil moisture conservation, shaping, cleaning.

Unit of measurement : plant

Quota number			1-172	1-173	1-174	1-175	1-176
Project			Plant shrubs (with soil balls) with soil ball diameter within (cm)				
			20	30	40	50	60
Name		Unit	Quantity				
Man-made	Combined work day	Work day	0. 046	0. 091	0. 137	0. 209	0. 382
Material	Water	m^3	0. 025	0. 025	0. 050	0. 075	0. 100

Note: When planting exceptionally large or rare trees, the cost shall be calculated separately.

Work content : Digging, cutting (falling pit, righting, returning soil, tamping, building water surround), watering, covering, soil moisture conservation, shaping, cleaning.

Unit of measurement : plant

Quota number			1-177	1-178	1-179	1-180	1-181
Project			Plant shrubs (with soil balls) with soil ball diameter within (cm)				
			70	80	100	120	140
Name		Unit	Quantity				
Man-made	Combined work day	Work day	0. 419	0. 637	1. 028	1. 502	2. 220
Material	Water	m^3	0. 125	0. 150	0. 300	0. 400	0. 500
Machinery	5-ton truck-mounted crane	Machine-team	0. 011	0. 017	0. 020	0. 028	0. 040
	4-ton truck	Machine-team	0. 020	0. 030	0. 034	0. 047	0. 068

Note: When planting exceptionally large or rare trees, the cost shall be calculated separately.

(3) uprooting shrubs (naked roots)

Job description: Excavation, pit removal, pruning, slurry application, centralized transportation, and backfilling of the pit. Unit of measurement: plant

Quota number			1−182	1−183	1−184	1−185
Project			Remove shrubs (bare roots) with crown height within (cm)			
			100	150	200	250
Name		Unit	Quantity			
Man-made	Combined work day	Work day	0. 027	0. 046	0. 091	0. 155

Note: When excavating exceptionally large or valuable trees, the cost shall be calculated separately.

(4) shrub planting (naked root)

Job responsibilities: Digging holes, planting (including straightening, backfilling, and tamping), watering, covering with soil, moisture retention, shaping, and cleaning.

Unit of measurement: plant

Quota number			1−186	1−187	1−188	1−189
Project			Planting shrubs (bare-root) with a crown height of (cm or less)			
			100	150	200	250
Name		Unit	Quantity			
Man-made	Combined work day	Work day	0. 036	0. 055	0. 100	0. 173
Material	Water	m^3	0. 025	0. 025	0. 050	0. 075

Note: When planting exceptionally large or rare trees, the cost shall be calculated separately.

4. Planting Bamboo

(1) Excavation of bamboo (scattered bamboo)

Job description: Excavation, wrapping, pit removal, pruning, centralized transportation, and backfilling of the pit. Unit of measurement: plant

Quota number			1−190	1−191	1−192	1−193	1−194
Project			The diameter at breast height of excavated bamboo is within (cm).				
			2	4	6	8	10
Name		Unit	Quantity				
Man-made	Combined work day	Work day	0. 027	0. 055	0. 082	0. 137	0. 228
Material	Straw rope	kg	0. 500	1. 000	1. 000	1. 500	1. 500

(2) Planting bamboo (scattered bamboo)

Job responsibilities: Digging holes, planting (excavating holes, straightening, backfilling, tamping, constructing water barriers), watering, covering with soil, moisture retention, and shaping
、 put in order 。 Unit of measurement: plant

Quota number			1−195	1−196	1−197	1−198	1−199
Project			Planting diameter of bamboo (in cm)				
			2	4	6	8	10
Name		Unit	Quantity				
Man-made	Combined work day	Work day	0. 036	0. 055	0. 082	0. 155	0. 246
Material	Water	m^3	0. 025	0. 038	0. 050	0. 075	0. 100

(3) Excavation of bamboo (clump bamboo)

Job description: Excavation, wrapping, pit removal, pruning, centralized transportation, and backfilling of the pit. Unit of measurement: cluster

Quota number			1-200	1-201	1-202	1-203	1-204	1-205
Project			The diameter of the root cluster of excavated bamboo is within (cm).					
			30	40	50	60	70	80
Name		Unit	Quantity					
Man-made	Combined work day	Work day	0.064	0.118	0.218	0.364	0.428	0.546
Material	Straw rope	kg	0.500	1.000	1.000	1.500	2.000	2.500
Machinery	5-ton truck-mounted crane	Machine-team	-	-	-	–	0.011	0.017
	4-ton truck	Machine-team	-	-	-	-	0.020	0.030

(4) Planting bamboo (clump bamboo)

Job responsibilities: Digging holes, planting (excavating holes, straightening, backfilling, tamping, constructing water barriers), watering, covering with soil, moisture retention, shaping、put in order 。

Unit of measurement: plant

Quota number			1-206	1-207	1-208	1-209	1-210	1-211
Project			The diameter of the root cluster in planted bamboo is within (cm).					
			30	40	50	60	70	80
Name		Unit	Quantity					
Man-made	Combined work day	Work day	0.064	0.109	0.264	0.309	0.355	0.410
Material	Water	m^3	0.025	0.038	0.050	0.075	0.100	0.100
Machinery	5-ton truck-mounted crane	Machine-team	–	–	–	–	0.011	0.017
	4-ton truck	Machine-team	–	–	–	–	0.020	0.030

5. Planting Hedges

(1) Planting a hedge (single row)

Job description: Digging trenches, transplanting seedlings, backfilling soil, constructing water barriers, watering, covering with soil, maintaining soil moisture, fertilizing, shaping, and cleaning.

Unit: 10m

Quota number			1-212	1-213	1-214	1-215	1-216	1-217
Project			Plant a single-row hedge (height within cm)					
			40	60	80	100	120	150
Name		Unit	Quantity					
Man-made	Combined work day	Work day	0. 473	0. 582	0. 764	1. 047	1. 256	1. 602
Material	Water	m³	0. 150	0. 200	0. 250	0. 300	0. 400	0. 500

(2) Planting hedges (double rows)

Job description: Digging trenches, transplanting seedlings, backfilling soil, constructing water barriers, watering, covering with soil, maintaining soil moisture, fertilizing, shaping, and cleaning.

Unit: 10m

Quota number			1-218	1-219	1-220	1-221
Project			Plant double-row hedges with height within cm			
			40	60	80	100
Name		Unit	Quantity			
Man-made	Combined work day	Work day	0. 582	0. 792	1. 028	1. 465
Material	Water	m³	0. 200	0. 250	0. 300	0. 400

6. Climbing Plant Cultivation

Job description: Digging trenches, transplanting seedlings, backfilling soil, constructing water barriers, watering, covering with soil, maintaining soil moisture, fertilizing, shaping, and cleaning.

Unit of measurement: 100 units

Quota number			1-222	1-223	1-224	1-225
Project			Climbing plant cultivation			
			Third year	Fourth grade	Fifth grade	Born in 1968-1988
	Name	Unit	Quantity			
Man-made	Combined work day	Work day	0. 846	1. 128	2. 603	4. 414
Material	Water	m^3	1. 320	1. 430	1. 650	1. 870
	Fertilizer	Kg	5. 500	5. 500	5. 500	5. 500

7. Planting Strip

Work **contents: digging holes, planting, scattering seedlings, pruning, coating with preservatives, planting, compacting the soil, opening the embankment, bundling the pillars,**watering and cleaning. Unit: $10m^2$

Quota number			1-226	1-227	1-228	1-229
Project			Strip height (in meters)			
			0. 8	1. 2	1. 5	1. 8
	Name	Unit	Quantity			
Man-made	Combined work day	Work day	0. 119	0. 209	0. 273	0. 346
Material	Water	m^3	0. 330	0. 330	0. 495	0. 495

8. Planting Flowers

Job description: Soil preparation, debris removal, base fertilizer application, land surveying, planting, watering, and cleanup.

Unit of measurement: 10m ²

Quota number			1-230	1-231	1-232	1-233	1-234
Project			Open-field flower planting				
			Herbal flowers	Wooden Flower	Corynbium, Rhizomata	General pattern flower bed	Painted pattern flower bed
Name		Unit	Quantity				
Man-made	Combined work day	Work day	1. 265	0. 983	1. 110	1. 847	2. 366
Material	Water	m^3	0. 500	0. 250	0. 300	0. 500	0. 500
	Manure	m^3	0. 125	0. 063	0. 110	0. 350	0. 350
	Flower seedling	Stock	(250. 000)	(63. 000)	(110. 000)	(700. 000)	(700. 000)

Job description: Soil preparation, debris removal, base fertilizer application, land surveying, planting, watering, and cleanup.

Unit of measurement: 10m ²

Quota number			1-235	1-236
Project			Open-field flower planting	
			A flower bed with a general pattern of five-colored grass	Five-color grass pattern flower bed
Name		Unit	Quantity	
Man-made	Combined work day	Work day	4. 259	5. 096
Material	Water	m^3	0. 500	0. 500
	Manure	m	0. 400	0. 400
	Flower seedling	Stock	(4000. 000)	(4000. 000)

9. Planting Aquatic Plants

Job description: dredging, transporting, planting and maintaining. Unit of measurement: 10 strains

Quota number			1-237	1-238
Project			Planting aquatic plants	
			Lotus	Lotus
Name		Unit	Quantity	
Man-made	Combined work day	Work day	0. 455	2. 448
Material	Clay jar	Single	-	10. 000
	Manure	m	0. 100	0. 100
	Flower seedling	stock	(10. 000)	(10. 000)

10. Floral Arrangement

Job content: turning the soil, clearing debris, applying base fertilizer, laying out, planting, watering, and cleaning. Measurement unit: 10m 2

Quota number			1-239	1-240
Project			Open-field flower planting	
			Three-dimensional flower bed	Five-color Grass Three-dimensional Flower Bed
Name		Unit	Quantity	
Man-made	Combined work day	Work day	3. 504	6. 689
Material	Water	m^3	0. 500	0:500
	Flower seedling	Stock	(700. 000)	(5000. 000)

11. Laying Turf

Job description: Turn the soil, clear debris, move and lay turf, water, and clean. Unit of measurement: $10m^2$

Quota number			1-241	1-242	1-243	1-244
Project			Laying turf			
			Scatter	Full coverage	Orthoband	Sow seeds
Name		Unit	Number Quantity			
Man-made	Combined work day	Work day	1. 229	1. 684	1. 110	0. 883
Material	Turf	m^2	3. 300	11. 000	10. 000	0. 200
	Water	m^3	0. 500	0. 500	0. 500	0. 200

Job duties: laying and compacting, installing flower grid grooves, watering, soil covering, and on-site transportation.

Unit of measurement: $10m^2$

Quota number			1-245	1-246
Project			The curved surface is fully covered with turf.	Herringbone turf
Name		Unit	Quantity	
Man-made	Combined work day	Work day	1. 765	0. 430
Material	Turf	m^2	11. 000	3. 700
	Water	m^3	0. 525	0. 525

12. Spray Seeding

Job description: Manual slope finishing, spraying and covering on shady slopes, and fixing.

Unit of measurement: 100m^2

Quota number			1-247	1-248	1-249	1-250	1-251	1-252
Project			Slope below 1:1			Slope ratio 1:1 or higher		
			Within 8 meters	Within 12 meters	12 meters away	Within 8 meters	Within 12 meters	12 meters away
Name		Unit	Quantity					
Human being	Combined work day	Work day	3. 485	4. 131	4. 532	4. 213	4. 614	4. 696
Material	Spray water retention agent	Kg	0. 400	0. 400	0. 500	0. 400	0. 400	0. 500
	Spray binder	Kg	0. 130	0. 140	0. 150	0. 130	0. 140	0. 150
	Non-woven fabrics	m^2	120. 000	120. 000	120. 000	120. 000	120. 000	120. 000
	Water	m^3	8. 000	9. 000	10. 000	8. 000	9. 000	10. 000
	Compound fertilizer	Kg	0. 600	0. 700	0. 800	0. 600	0. 700	0. 800
	Seeds (Comprehensive)	Kg	2. 500	2. 500	3. 500	2. 500	3. 500	3. 500
Machinery	4-ton truck	Machine-team	0. 096	0. 059	0. 070	0. 098	0. 063	0. 075
	Sprayer 2.5t	Machine-team	0. 096	–		0. 096	-	
	Sprayer 3t	Machine-team	_	0. 059	–	_	0. 063	–
	3.5t Sprayer Other	Machine-team	–	–	0. 070	-	-	0. 075
	Machinery Costs	Yuan	2. 830	3. 360	3. 680	3. 420	3. 750	3. 820

Job description: Soil preparation, debris removal, sowing, and watering.

Unit of measurement: 10m^2

Quota number			1-253
Project			Artificial mixed turf
Name		Unit	Quantity
Man-made	Combined work day	Work day	3. 194
Material	Grass seed	Kg	0. 300
	Water	m^3	0. 500
	Manure	m^3	0. 400

13. Planting Grass in Brick

Job description: Hole cleaning, planting soil filling, planting, etc. Unit of measurement: $10m^2$

Quota number			1-254
Project			Grass planting in brick holes
Name		Unit	Quantity
Man-made	Combined work day	Work day	0. 513
Material	Planting soil	m^3	0. 300
	Water	m^3	0. 367
	Fertilizer	Kg	0. 367
	Other material costs	Yuan	0. 050

14. Potting

(1) artificial tree (naked root)

Job description: Digging holes, planting seedlings, spreading seedlings, pruning, applying preservatives, planting, compacting soil, creating furrows, bundling support posts, watering, and cleaning. Unit of measurement: plant

Quota number			1-255	1-256	1-257	1-258
Project			The diameter at breast height (DBH) of the planted tree (naked root) is within (cm).			
			4	6	8	10
Name		Unit	Quantity			
Man-made	Combined work day	Work day	0. 130	0. 212	0. 290	0. 368
Material	Bambus clasp	Root	1. 100	1. 100	1. 100	1. 100
	Water	m^3	0. 330	0. 495	0. 660	0. 660

Job description: Digging holes, planting seedlings, spreading seedlings, pruning, applying preservatives, planting, compacting soil, creating furrows, bundling support posts, watering

Water and site cleanup. Unit of measurement: plant

Quota number			1-259	1-260	1-261	1-262
Project			The diameter at breast height (DBH) of the planted tree (naked root) is within (cm).			
			12	15	20	25
Name		Unit	Quantity			
Man-made	Combined work day	Work day	0. 824	1. 108	1. 481	1. 881
Material	Bambus clasp	Root	1. 100	1. 100	1. 100	1. 100
	Water	m³	0. 825	0. 990	1. 155	1. 320

(2) false shrub (naked root)

Job description: Digging holes, planting seedlings, spreading seedlings, pruning, applying preservatives, planting, compacting soil, creating furrows, bundling support posts, watering

Water and site cleanup. Unit of measurement: plant

Quota number			1-263	1-264	1-265	1-266
Project			The height of the crown of the planted shrub (bare-root) is within (cm).			
			100	150	200	250
Name		Unit	Quantity			
Man-made	Combined work day	Work day	0. 090	0. 119	0. 154	0. 200
Material	Water	m³	0. 330	0. 330	0. 495	0. 660

(3) Boxed Seedlings

Job responsibilities: Digging holes, planting seedlings, spreading seedlings, pruning, applying preservatives, planting, compacting soil, creating furrows, bundling support posts, and cleaning.

texture 。 Unit of measurement: plant

Quota number			1-267	1-268	1-269
Project			Boxed seedlings (box size in cm)		
			200×200×90	250×250×100	300×300×110
Name		Unit	Quantity		
Man-made	Combined work day	Work day	10. 100	16. 090	23. 170
Material	Finished wooden box	Cover	(1. 000)	(1. 000)	(1. 000)
	with iron core	Root	110. 000	118. 000	138. 000
	Iron nail [round nail]	kg	13. 036	15. 045	17. 595
	large herb slice	slice	20. 000	28. 000	34. 000
	Other material costs	Yuan	12. 400	14. 270	16. 930
Machinery	5t truck crane,	Machine-team	0. 100	0. 160	0. 230
	8t truck	Machine-team	0. 330	-	-
	10-ton truck	Machine-team	–	0. 400	0. 500

15. Planting Arrangement

Job duties: manual loading and unloading, layout, flower arrangement, flower removal, and cleaning. Unit of measurement: 100 basins

Quota number			1-270	1-271	1-272	1-273
Project			Potted flower arrangement			
			Pelvic diameter (within cm with pattern) plane		Pelvic diameter (within cm with patterned design) oblique surface	
			20	30	20	30
Name		Unit	Quantity			
Man-made	Combined work day	Work day	0. 700	0. 960	1. 000	1. 200

Section 3: Sprinkler Irrigation in Green Spaces (050103)

I

Explain

1. This section includes 137 items, such as the installation of sprinkler pipelines and accessories, the assembly and installation of water meters, sprinkler nozzles, the installation of quick-release water valves, the oiling of pipes and iron fittings, the masonry of wells, the installation of automatic intelligent sprinkler systems, and the installation of fixed ratio fertilization systems.

2. This quota applies to all pipeline installations, whether surface-mounted or directly buried underground.

3. For surface-mounted pipelines requiring metal fittings, their standalone value shall be calculated separately, while installation costs and operational losses are excluded from separate calculation. 4. Earthwork for underground direct-buried pipelines shall be subject to the corresponding sub-items in Chapter IV of this quota under General Items.

5. If the masonry and installation of solenoid valves or valve boxes differ from those specified in this subsection, separate conversion shall apply.

6. The automatic intelligent sprinkler system is excluded from the central control system and control software costs, which will be calculated separately when incurred.

7. For sprinkler irrigation installation projects requiring commissioning, the commissioning fee shall be 1% of the total project cost. For three-dimensional flower bed sprinkler irrigation installation projects, the commissioning fee shall be 1.5% of the total project cost.

8. This section excludes the erection and dismantling of construction scaffolding and mobile scaffolding, with corresponding costs to be calculated separately when applicable.

Rules for Calculation of Quantity of Work

1. Pipeline installation shall be calculated based on the centerline length shown in the design drawings, without deducting the length occupied by valves, pipe fittings, and accessories. For vertical flower bed pipelines, the pipe splicing length at the disassembly point shall be calculated according to the drawing requirements. Water supply pipe installation shall be calculated based on the quantity shown in the design drawings.

2. Water meters are categorized by specifications and connection types, with quantities calculated accordingly.

3. In the three-dimensional sprinkler and drip irrigation system, the installation of drip nozzles and sprinklers is calculated by quantity; the installation of subsurface irrigation systems is calculated by length; the installation of adjustable PE connecting pipes for sprinklers is calculated by length.

4. The oiling diameter of pipelines is calculated by length, while that of iron parts is calculated by mass.

5. The sub-pressure, specifications, and connection methods of valves shall be calculated based on quantity; the specifications of solenoid valves and valve boxes shall be calculated based on quantity; automatic intake/exhaust valves and drain valves shall be calculated based on quantity.

6. The types and specifications of nozzles, quick-release water valves, and root irrigators shall be calculated based on quantity.

7. Well body masonry is calculated by quantity according to its form; manhole cover installation is also calculated by quantity.

8. The sensing device, controller, module, decoder, and grounding electrophoresis protector shall be quantified separately according to different measurement units.

9. The fixed ratio fertilization system is calculated by quantity according to specifications.

1. Sprinkler Pipeline

(1) Galvanized steel pipe (threaded connection)

Work content : on-site handling, inspection, cleaning pipe, cutting pipe, sleeve wire parts, straightening pipe, steel installation and hydraulic test.

measurement unit： m

Quota number			1-274	1-275	1-276	1-277	1-278	1-279
Project			Nominal diameter (within mm)					
			15	20	25	32	40	50
Name		Unit	Quantity					
Human being	Combined work day	Work day	0. 054	0. 054	0. 062	0. 062	0. 067	0. 078
Material	Galvanized steel pipe DN20	m	–	1. 015	–	–	–	–
	Galvanized steel pipe DN25	m	–	–	1. 015	–	–	–
	Galvanized steel pipe DN32	m	–	–	–	1. 015	–	–
	Galvanized steel pipe DN40	m	–	–	–	–	1. 015	–
	Galvanized steel pipe DN50	m	–	–	–	–	–	1. 015
	Galvanized steel pipe DN15	m	0. 150	–	–	–	–	–
	Engine oil	kg	0. 002	0. 003	0. 003	0. 003	0. 004	0. 004
	Outdoor galvanized steel pipe joint parts DN15	one	0. 190	–	–	–	–	–
	Outdoor galvanized steel pipe joint parts DN20	one	–	0. 192	–	–	–	–
	Outdoor galvanized steel pipe joint parts DN25	one	–	–	0. 192	–	–	–
	Outdoor galvanized steel pipe joint parts DN32	one	–	–	–	0. 192	–	–

Continue Sheet

Quota number			1-274	1-275	1-276	1-277	1-278	1-279
Project			Nominal diameter (within mm)					
			15	20	25	32	40	50
Name		Unit	Quantity					
Material	Outdoor galvanized steel pipe joint parts DN40	Individual	–	–	–	–	0. 186	–
	Outdoor galvanized steel pipe joint parts DN50	Individual	–	–	–	–	–	0. 185
	Electricity	kw • h	–	–	0. 012	0. 015	0. 015	0. 018
	Water	m^3	0. 005	0. 006	0. 008	0. 010	0. 013	0. 016
	Other material costs	Yuan	0. 160	0. 190	0. 250	0. 330	0. 390	0. 480
Machinery	Grinding wheel cutter for pipes up to Φ500, threading machine for pipes up to Φ159, and other machinery costs	Machine-team	–	–	0. 001	0. 001	0. 001	0. 001
		Machine-team	–	–	0. 002	0. 003	0. 003	0. 004
		Yuan	0. 170	0. 170	0. 190	0. 200	0. 210	0. 250

Job responsibilities: On-site material handling, inspection and cleaning of piping, pipe cutting, threading and fitting, straightening of pipes, steel component installation, and hydraulic testing, etc.   measurement unit：m

Quota number			1-280	1-281	1-282
Project			Nominal diameter (within mm)		
			70	80	100
	Name	Unit	Quantity		
Human being	Combined work day	Work day	0. 084	0. 090	0. 108
Material	Galvanized steel pipe DN70	m	1. 015	–	–
	Galvanized steel pipe DN80	m	–	1. 015	–
	Galvanized steel pipe DN100	m	–	–	1. 015
	Engine oil	kg	0. 003	0. 003	0. 003
	Outdoor galvanized steel pipe joint parts DN70	one	0. 176	–	–
	Outdoor galvanized steel pipe joint parts DN80	one	–	0. 172	–
	Outdoor galvanized steel pipe joint parts DN100	one	–	–	0. 163
		kw·h	0. 022	0. 027	0. 072
	Water	m^3	0. 022	0. 025	0. 031
	Other material costs	yuan	0. 640	0. 800	1. 020
Machinery	Pipe cutting thread machine Φ159, grinding wheel cutter Φ500 and other machinery costs	Machine-team	0. 002	0. 002	0. 002
		Machine-team	0. 003	0. 004	0. 130
		Yuan	0. 260	0. 280	0. 340

(2) PE pipe

① underground

Job responsibilities: On-site material handling, visual inspection, pipe cutting, hot-melt welding, pipe fitting installation, pipe straightening, and initial hydraulic testing.

measurement unit：m

Quota number			1-283	1-284	1-285	1-286	1-287	1-288
Project			Pipe diameter (in mm)					
			20	25	32	40	50	63
	Name	Unit	Quantity					
Man-made	Combined work day	Work day	0. 047	0. 047	0. 058	0. 058	0. 073	0. 091
Material	PE pipe, Φ20	m	1. 020	–	–	–	–	–
	PE pipe fitting Φ20	one	0. 284	–	–	–	–	–
	PE pipe, Φ25	m	–	1. 020	–	–	–	–
	PE pipe fitting Φ25	one	–	0. 284	–	–	–	–
	PE pipe, Φ32	m	–	–	1. 020	–	–	–
	PE pipe fitting Φ32	one	–	–	0. 268	–	–	–
	PE pipe, Φ40	m	–	–	–	1. 020	–	–
	PE pipe fitting Φ40	one	–	–	–	0. 268	–	–
	PE pipe, Φ50	m	–	–	–	–	1. 020	–
	PE pipe fitting Φ50	one	–	–	–	–	0. 226	–
	PE pipe, Φ63	m	–	–	–	–		1. 020
	PE pipe fitting Φ63;	one	–	–	–	–		0. 192
	Other material costs	yuan	0. 070	0. 090	0. 170	0. 250	0. 330	0. 530
Machinery	Other machinery costs	Yuan	0. 150	0. 150	0. 180	0. 180	0. 230	0. 290

② Embedded and Spliced Within the Three-Dimensional Floral Skeleton

Job responsibilities: On-site material handling, visual inspection, pipe cutting, adhesive application, pipe fitting installation, straightening, and initial hydraulic testing.

measurement unit： m

Quota number			1-289	1-290	1-291	1-292
Project			Pipe diameter (in mm)			
			16	20	25	32
Name		Unit	Quantity			
Man-made	Combined work day	Work day	0. 143	0. 143	0. 129	0. 129
Material	PE pipe Φ16	m	1. 020	–		–
	PE pipe fitting Φ16	one	1. 326	–	–	–
	PE pipe, Φ20	m	–	1. 020	–	–
	PE pipe fitting Φ20	one		1. 326	–	–
	PE pipe, Φ25	m	–	–	1. 020	–
	PE pipe fitting Φ25	one	–	–	1. 326	–
	PE pipe, Φ32	m	–	–	–	1. 020
	PE pipe fitting Φ32	one			–	1. 326
	Nylon tie L=100~150	root	1. 010	1. 010	1. 010	1. 010
	Other material costs	yuan	0. 140	0. 180	0. 220	0. 370
Machinery	Other machinery costs	Yuan	0. 450	0. 450	0. 410	0. 410

(3) Water Pipe Consolidation

Job responsibilities: On-site cleanup, concrete pouring, vibration, and protection of the structure. Unit of measurement: see table

Quota number			1-293	1-294	1-295
Project			Up to Φ75	Within Φ100	Filler sand
			Dwell		m^2
Name		Unit	Quantity		
Man-made	Combined work day	Work day	0. 010	0. 010	0. 200
Material	Sand	m^3	–	–	1. 002
	Ready-mixed concrete C20	m^3	0. 034	0. 060	–
	Other material costs	Yuan	0. 190	0. 340	1. 790
Machinery	Insertion type concrete vibrator	Machine-team	0. 030	0. 030	–
	Other machinery costs	Yuan	0. 030	0. 030	0. 770

2. Sprinkler Fittings Installation

(1) Low Pressure Threaded Valve

Job responsibilities: On-site material handling, visual inspection, rust removal, straightening, valve installation, and hydraulic testing.

Unit of measurement: pieces

Quota number			1-296	1-297	1-298	1-299	1-300	1-301
Project			Nominal diameter (within mm)					
			15	20	25	32	40	50
Name		Unit	Quantity					
Human being	Combined work day	Work day	0.035	0.040	0.050	0.055	0.080	0.090
Material	Valve	Indi-	(1.000)	(1.000)	(1.000)	(1.000)	(1.000)	(1.000)
	Rubber plate δ 1~3	vidual	0.002	0.003	0.004	0.006	0.008	0.010
	Engine oil	kgkg	0.012	0.012	0.012	0.012	0.016	0.016
	Live joint pad 15	One, one, one, one, one, one	1.050	–	–	–	–	–
	Live joint pad 20		–	1.050	–	–	–	–
	Live joint pad 25		–	–	1.050	–	–	–
	Live joint pad 32		–	–	–	1.050		–
	Live joint pad 40		–	–	–	–	1.050	–
	Live joint pad 50		–	–	–	–		1.050

Continue Sheet

Quota number			1-296	1-297	1-298	1-299	1-300	1-301
Project			Nominal diameter (within mm)					
			15	20	25	32	40	50
Name		Unit	Quantity					
Material	Galvanized welded joint DN15	one	1.010	–	–	–	–	–
	Galvanized welded joint DN20	one	–	1.010	–	–	–	–
	Galvanized welded joint DN25	one	–	–	1.010	–	–	–
	Galvanized welded joint DN32	one	–	–	–	1.010	–	–
	Galvanized welded joint DN40	one	–	–	–	–	1.010	–
	Galvanized welded joint DN50	one	–	–	–	–	–	1.010
	Electricity	kw·h	0.005	0.010	0.015	0.025	0.030	0.035
	Other material costs	Yuan	0.630	0.760	0.890	1.050	1.220	1.400
Machinery	Other machinery costs for grinding wheel cutters up to Φ500	Tian-ban	0.001	0.002	0.003	0.005	0.006	0.007
		Yuan	0.110	0.130	0.160	0.170	0.250	0.280

(2) Low Pressure Threaded Flange Valve

Job responsibilities: On-site material handling, surface inspection, rust removal, flange installation, gasket fabrication, and hydraulic testing.

Unit of measurement: pieces

Quota number			1-302	1-303	1-304	1-305
Project			Nominal diameter (within mm)			
			50	70	80	100
Name		Unit	Quantity			
Human being	Combined work day	Work day	0. 326	0. 492	0. 605	1. 204
Material	Valve	one	(1. 000)	(1. 000)	(1. 000)	(1. 000)
	paronite δ 3	kg	0. 140	0. 180	0. 260	0. 350
	engine oil	kg	0. 020	0. 020	0. 020	0. 024
	Steel washer M16	one	8. 240	8. 240	8. 240	16. 480
	Mother-of-pearl bolt M12×65~80	series	–	8. 240	8. 240	16. 480
	Mother-of-pearl bolt M16×65~80	series	8. 240	–	–	
	Threaded flange (below 1.0Mpa) 50	piece	2. 000	–		–
	Threaded flange (below 1.0Mpa) 70	piece	–	2. 000	–	–
	Threaded flange (below 1.0Mpa) 80	piece	–	–	2. 000	–
	Threaded flange (below 1.0 Mpa) 100 units		–	–	–	2. 000
		kw·h	0. 035	0. 045	0. 050	0. 080
	Other material costs	Yuan	3. 420	4. 200	4. 850	4. 530
Machinery	Other machinery costs for grinding wheel cutters up to Φ 500	Tia-nban	0. 007	0. 009	0. 010	0. 016
		Yuan	1. 030	1. 550	1. 900	3. 790

(3) Valve With Welded Plastic Flange

Job **content: on-site handling, appearance inspection, flange pad making and installation, valve and flange installation, hydraulic test, etc.**

Unit of measurement: pieces

Quota number			1-306	1-307	1-308	1-309	1-310	1-311	1-312	1-313
Project			Pipe diameter (in mm)							
			20	25	32	40	50	70	80	100
Name		Unit	Quantity							
Man-made	Combined work day	Work day	0. 205	0. 267	0. 271	0. 282	0. 289	0. 393	0. 400	0. 479
Material	Plastic flange (with bolts)	1 piece	(2. 000)	(2. 000)	(2. 000)	(2. 000)	(2. 000)	(2. 000)	(2. 000)	(2. 000)
	Valve		(1. 000)	(1. 000)	(1. 000)	(1. 000)	(1. 000	(1. 000)	(1. 000)	(1. 000)
	Acid resistant asbestos rubber sheet	kg	0. 060	0. 070	0. 080	0. 110	0. 140	0. 180	0. 260	0. 350
	Plastic welding rod	kg kw	0. 010	0. 020	0. 020	0. 020	0. 030	0. 050	0. 060	0. 080
	Electricity	h •	0. 840	1. 035	1. 110	1. 125	1. 725	2. 055	2. 385	2. 850
	Other material costs	Yuan	0. 550	0. 610	0. 660	0. 880	1. 110	1. 640	2. 190	2. 740
Machinery	Electric air compressor 0.6m^3/min	Tian-ban	0. 056	0. 069	0. 074	0. 075	0. 115	0. 137	0. 159	0. 190
	Other machinery costs	Yuan	0. 650	0. 840	0. 850	0. 890	0. 910	1. 240	1. 260	1. 510

(4) Low Pressure Plastic Valve

Job responsibilities: On-site material handling, visual inspection, valve installation, hydraulic testing, etc. Unit of measurement: pieces

Quota number			1-314	1-315	1-316	1-317	1-318
Project			Pipe diameter (in mm)				
			20	25	32	40	50
Name		Unit	Quantity				
Man-made	Combined work day	Work day	0.076	0.091	0.111	0.143	0.241
Material	Valve	One, one, one, one, one, one yuan	(1.000)	(1.000)	(1.000)	(1.000)	(1.000)
	Socketed plastic pipe fitting 20		2.020	–	–	–	–
	Socketed plastic pipe fitting 25		– –	2.020	–	–	–
	Socketed plastic pipe fitting 32		– –	–	2.020	–	–
	Socketed plastic pipe fitting 40		–	–	–	2.020	–
	Socketed plastic pipe fitting 50		–	–	–	–	2.020
	Other material costs		0.650	0.730	0.850	1.050	1.260
Machinery	Other machinery costs	Yuan	0.240	0.290	0.350	0.450	0.760

(5) Flanged Welded Valve

Job responsibilities: On-site material handling, surface inspection, rust removal, welding, flange installation, gasket fabrication, and valve/flange assembly.

The bolt fastening and the permanent pressure test are carried out. Unit of measurement: pieces

Quota number			1-319	1-320	1-321	1-322
Project			Nominal diameter (within mm)			
			50	70	80	100
Name		Unit	Quantity			
Human being	Combined work day	Work day	0.293	0.362	0.370	0.498
Material	Valve	one	(1.000)	(1.000)	(1.000)	(1.000)
	Rubber gasket 16	one	8.240	8.240	8.240	16.480
	asbestos rubber plate δ3	kg	0.140	0.180	0.260	0.294
	welding rod	kg	0.133	0.237	0.271	0.372
	Acetylene gas	m^3	0.003	0.024	0.030	0.038
	Mother bolt M16×65~80	series	8.240	8.240	8.240	16.480
	flat-welded flange (for pressures below 1.0Mpa),50	piece	2.000	–	–	–
	Flat-welded flange (below 1.0Mpa) 70	piece	–	2.000	–	–
	Flat-welded flange (below 1.0Mpa) 80	piece	–	–	2.000	–
	Flat-welded flange (below 1.0 Mpa) 100	piece		–		2.000
	electricity	kw·h	2.351	2.918	3.369	4.198
	Other material costs	Yuan	2.790	3.820	4.750	6..150
Machinery	for welding machines up to 40kVA	Machine-team	0.077	0.097	0.112	0.139
	for welding rod drying box	Tianban	0.006	0.008	0.009	0.010
	Other mechanical costs	Yuan	0.920	1.140	1.160	1.570

(6) Solenoid Valve, Valve Box

Job **content: on-site handling, appearance inspection, thread connection, wiring protection, waterproof joint, foundation masonry, valve box** installation, etc. Unit of measurement: pieces

<table>
<tr><td colspan="3">Quota number</td><td>1-323</td><td>1-324</td><td>1-325</td><td>1-326</td><td>1-327</td><td>1-328</td><td>1-329</td></tr>
<tr><td colspan="3" rowspan="2">Project</td><td colspan="7">Diameter (mm)</td></tr>
<tr><td>20</td><td>25</td><td>32</td><td>40</td><td>50</td><td>80</td><td>100</td></tr>
<tr><td colspan="2">Name</td><td>Unit</td><td colspan="7">Quantity</td></tr>
<tr><td>Man-made</td><td>Combined work day</td><td>Work day</td><td>0. 092</td><td>0. 110</td><td>0. 138</td><td>0. 188</td><td>0. 229</td><td>0. 550</td><td>0. 734</td></tr>
<tr><td>Material</td><td>Waterproof
joint for so-
lenoid valve
UPVC external thread joint 20
UPVC external thread joint 25
UPVC external thread joint 32
UPVC external thread joint 40
UPVC external thread joint 50
UPVC external thread joint 80
UPVC external thread joint
100, UPVC elbow 20
UPVC elbow 25
UPVC elbow 32
UPVC elbow 40
UPVC elbow 50
UPVC elbow 80
UPVC elbow
100, drain valve
Φ25, and other
material costs</td><td>one
one
one
one
one
one
one
one
one
one
one
one
one
one
one
one
one
one
one
one
one
one
one
one
yuan</td><td>(1. 000)
2. 000
2. 000
2. 000
–
–
–
-
-
2. 000
-
-
-
-
1. 000
2. 270</td><td>(1. 000)
2. 000
2. 000
2. 000
-
-
-
-
2. 000
-
-
-
1. 000
2. 300</td><td>(1. 000)
2. 000
2. 000
–
2. 000
-
–
–
-
2. 000
–
–
1. 000
2. 330</td><td>(1. 000)
2. 000
2. 000
–
–
2. 000
-
-
–
–
2. 000
–
–
1. 000
2. 600</td><td>(1. 000)
2. 000
2. 000
-
–
–
2. 000
-
–
–
2. 000
1. 000
2. 770</td><td>(1. 000)
2. 000
2. 000
–
-
–
2. 000
–
–
–
–
–
2. 000
1. 000
4. 200</td><td>(1. 000)
2. 000
2. 000
–
–
–
–
–
2. 000
–
–
–
–
–
2. 000
1. 000
5. 580</td></tr>
<tr><td>Machinery</td><td>Other machinery costs</td><td>Yuan</td><td>0. 290</td><td>0. 350</td><td>0. 430</td><td>0. 580</td><td>0. 720</td><td>1. 730</td><td>2. 310</td></tr>
</table>

Job responsibilities: On-site material handling, exterior inspection, threaded connections, wiring protection, waterproof joints, foundation masonry, and valve box installation.

etc. Unit of measurement: pieces

<table>
<tr><td colspan="3">Quota number</td><td>1-330</td><td>1-331</td><td>1-332</td><td>1-333</td></tr>
<tr><td colspan="3" rowspan="3">Project</td><td colspan="2">Valve box (round)</td><td colspan="2">Square valve box</td></tr>
<tr><td rowspan="2">No foundation</td><td rowspan="2">Basic</td><td>536×403×30
0mm</td><td>654×476×30
0mm</td></tr>
<tr><td colspan="2">Basic</td></tr>
<tr><td colspan="2">Name</td><td>Unit</td><td colspan="4">Quantity</td></tr>
<tr><td>Man-made</td><td>Combined work day</td><td>Work day</td><td>0. 088</td><td>0. 363</td><td>0. 367</td><td>0. 375</td></tr>
<tr><td rowspan="4">Material</td><td>Clack box</td><td>Individual</td><td>(1. 000)</td><td>(1. 000)</td><td>(1. 000)</td><td>(1. 000)</td></tr>
<tr><td>Cement mortar M7.5-S-3</td><td>m</td><td>_</td><td>0. 022</td><td>0. 048</td><td>0. 064</td></tr>
<tr><td>Shale brick</td><td>block</td><td>–</td><td>43. 260</td><td>92. 700</td><td>123. 600</td></tr>
<tr><td>Other material costs</td><td>yuan</td><td>0. 510</td><td>0. 960</td><td>1. 480</td><td>1. 800</td></tr>
<tr><td>Machinery</td><td>Other machinery costs</td><td>Yuan</td><td>0. 280</td><td>1. 140</td><td>1. 160</td><td>1. 180</td></tr>
</table>

(7) Automatic Intake Valve, Drain Valve

Job responsibilities: On-site material handling, visual inspection, and threaded connection operations. Unit of measurement: pieces

Quota number			1-334	1-335	1-336
Project			Nominal diameter (mm)		
			20	25	50
Name		Unit	Quantity		
Man-made	Combined work day	Work day	0.091	0.100	0.138
Material	Inlet and exhaust valve DN20	one	(1.000)	–	–
	Inlet and exhaust valve DN25	one	–	(1.000)	–
	Inlet and exhaust valve DN50	one	–	–	(1.000)
	UPVC tee 20	one	1.010	–	–
	UPVC tee 25	one	–	1.010	–
	UPVC tee 50	one	–		1.010
	UPVC external thread joint 20	one	1.000	–	–
	UPVC external thread joint 25	one	–	1.000	–
	UPVC external thread joint 50	one	–	–	1.000
	Threaded ball valve Φ20	one	1.000	–	–
	Threaded ball valve Φ25	one	–	1.000	–
	Threaded ball valve Φ50	one	–	–	1.000
	Drain valve Φ20	one	1.000	–	–
	Drainage valve Φ25	one	–	1.000	–
	Drainage Valve Φ50	one	–	–	1.000
	Other Material Costs	yuan	0.930	1.260	1.960
Machinery	Other machinery costs	Yuan	0.290	0.310	0.430

3. Composition and Installation of Water Meter

(1) Threaded Connection

Job responsibilities: On-site material handling, cleaning and inspection, installation of pads, leveling, alignment, valve installation, water meter installation, water supply testing, etc.

Unit of measurement: group

Quota number			1-337	1-338	1-339	1-340	1-341	1-342
Project			Nominal diameter (within mm)					
			15	20	25	30	40	50
Name		Unit	Quantity					
Human being	Combined work day	Work day	0.291	0.342	0.410	0.479	0.581	0.684
Material	Water meter	Block	(1.000)	(1.000)	(1.000)	(1.000)	(1.000)	(1.000)
	Rubber plate δ 1~3	kg	0.050	0.050	0.050	0.050	0.050	0.050
	Engine oil	kg	0.010	0.010	0.010	0.010	0.010	0.010
	Steel swivel joint 15	cover	2.000	–	–	–	–	–
	Steel flexible joint 20	Cover	–	2.000	–	–	–	–
	Steel flexible joint 25	Cover	–	–	2.000	–	–	–
	Steel swivel joint 32	Cover	–	–	–	2.000	–	–
	Steel swivel joint 40	Cover	–	–	–	–	2.000	–
	Steel flexible joint 50	Cover	–	–	–	–	–	2.000
	Threaded globe valve J11T-16 DN15	Individual	1.000	–	–	–	–	–
	Threaded globe valve J11T-16 DN20	Individual	–	1.000	–	–	–	–
	Threaded globe valve J11T-16 DN25	Individual	–	–	1.000	–	–	–

Continue Sheet

Quota number			1-337	1-338	1-339	1-340	1-341	1-342
Project			Nominal diameter (within mm)					
			15	20	25	30	40	50
Name		Unit	Quantity					
Material	Threaded globe valve J11T-16 DN32	one	–	–	–	1. 000	–	–
	Threaded globe valve J11T-16 DN40	one	–	–	–	–	1. 000	–
	Threaded globe valve J11T-16 DN50	one	–	–	–	–	–	1. 000
	Other material costs	yuan	1. 050	1. 270	1. 870	1. 940	2. 480	3. 340
Machin-ery	Other machinery costs	Yuan	0. 920	1. 080	1. 290	1. 510	1. 830	2. 150

(2) Flange Water Meter Composition and Installation (with Bypass Pipe and Check Valve)

Job **content: on-site handling, cleaning and inspection, welding, making and adding pads, water meter, valve installation,**bolt, hydraulic test, etc. Unit of measurement: group

Quota number			1-343	1-344	1-345
Project			Nominal diameter (within mm)		
			50	80	100
Name		Unit	Quantity		
Human being	Combined work day	Work day	2. 174	2. 430	2. 520
Material	Water meter	Block	(1. 000)	(1. 000)	(1. 000)
	Galvanized steel pipe DN50	m	1. 750	–	–
	Galvanized steel pipe DN80	m	–	2. 000	–
	Galvanized steel pipe DN80	m	–	–	2. 250
	Asbestos rubber board δ3	kg	0. 830	1. 570	2. 080
	welding rod	kg	1. 590	3. 810	4. 770
	Engine oil	kg	0. 900	0. 900	1. 200
	Acetylene gas	m^3	1. 400	0. 220	0. 260
	Gasket 16	one	49. 440	49. 440	98. 880
	DN80 compression elbow	one	–	2. 000	–
	DN100 compression elbow	one	–	–	2. 000
	DN50 compression elbow	one	2. 000	–	–

Continue Sheet

Quota number			1-343	1-344	1-345
Project			Nominal diameter (within mm)		
			50	80	100
	Name	Unit	Quantity		
Material	Mother bolt M16×65~80	Set	49.440	49.440	98.880
	Flat-welded flange (below 1.0Mpa) 5 0	piece	14.000	–	0.900
	Flat-welded flange (below 1.0Mpa) 80	piece	–	14.000	–
	Flat-welded flange (below 1.0Mpa) 1 0 0	piece	–	–	14.000
	Flanged check valve H44T-16-50	one	1.000	–	–
	Flanged check valve H44T-16-80	one	–	1.000	–
	Flanged check valve H44T-16-100	one	–	–	1.000
	Flanged gate valve Z44T-10-50	one	3.000	–	–
	Flanged gate valve Z44T-10-80	one	–	3.000	–
	Flanged gate valve Z44T-10-100	one	2	–	3.000
	Other material costs	yuan	25.280	35.370	45.030
Machin-ery	DC welding machine 20kW	Tian-ban	1.110	1.730	2.030
	other machinery costs	Yuan	6.840	7.650	7.930

4. Installation of Sprinkler Head and Quick Water Take-off Valve

(1) Nozzle, Quick-Release Water Valve

Job responsibilities: Installation and commissioning of nozzles and quick-release water valves. Unit of measurement: pieces

Quota number			1-346	1-347	1-348	1-349	1-350
Project			Blow head		Fixed sprinkler vertical pipe		
			Buried rotation, scattering	Switching rocker	6 meters tall	8 meters tall	12 meters high
	Name	Unit	Quantity				
Man-made	Combined work day	Work day	0.029	0.038	1.710	2.413	4.104
Material	Flat welding flange	Piece	–	–	(2.000)	(2.000)	(2.000)
	Adjustable nozzle	set	(1.000)	(1.000)	–	–	–
	Discharge valve	piece	–	–	(1.000)	(1.000)	(1.000)
	Tsuo hand-hole well	seat	–	–	(1.000)	(1.000)	(1.000)
	Galvanized steel pipe DN25	m	–	–	1.000	1.000	2.000
	Galvanized steel pipe DN32	m	–	–	1.500	1.500	2.500
	Galvanized steel pipe DN40	m	–	–	1.500	1.500	3.000
	Galvanized steel pipe DN50	m	–	–	2.000	2.000	3.000
	Galvanized steel pipe DN70	m	–	–	–	2.000	3.000
	UPVC threaded	one	1.000	1.000	–	–	–
	UPVC band	one	2.000	2.000	–	–	–
	Flanged gate valve Z44T-10-50	one	–	–	1.000	1.000	1.000
	ready-mixed concrete C20	m^3	–	–	0.120	0.120	0.150
	Other material costs	yuan	0.770	0.810	6.560	7.560	9.160
Machin-ery	Other machinery costs	Yuan	0.090	0.120	5.380	7.600	12.920

Job responsibilities: Installation and commissioning of nozzles and quick-release water valves. Unit of measurement: pieces

Quota number			1-351	1-352	1-353	1-354	1-355	1-356
Project			All copper water intake valve			Plastic water intake valve		
			20	25	50	20	25	50
Name		Unit	Quantity					
Human being	Combined work day	Work day	0. 169	0. 169	0. 440	0. 169	0. 169	0. 440
Material	Hot-rolled equal angle steel Q235>L63	kg	2. 798	2. 798	2. 798	2. 798	2. 798	2. 798
	wire joint	one	1. 000	1. 000	1. 000	1. 000	1. 000	1. 000
	DN50 clamp	one	1. 000	1. 000	1. 000	1. 000	1. 000	1. 000
	Quick Water Valve (Full Copper) DN20	one	1. 000	–	–	–	–	–
	Quick Water Valve (Full Copper) DN25	one	–	1. 000	–	–	–	–
	Quick-acting water valve (all-copper) DN50	one	–	–	1. 000	–	–	–
	Quick Water Valve (Plastic) DN20	one	–	–	–	1. 000	–	–
	Quick Water Valve (Plastic) DN25	one	–	–	–	–	1. 000	–
	Quick Water Valve (Plastic) DN50	one	–	–	–	–	–	1. 000
	– Other Material Costs	yuan	4. 950	6. 810	30. 560	2. 890	3. 850	5. 850
Machine	Other machinery costs	Yuan	0. 530	0. 530	1. 390	0. 530	0. 530	1. 390

Job responsibilities: On-site material handling, cleaning and inspection, welding, pad fabrication and installation, water meter installation, valve installation, bolt tightening, and water pressure testing

trial, etc. Unit of measurement: set

Quota number			1-357
Project			Root irrigator (tree cage)
Name		Unit	Quantity
Man-made	Combined work day	Work day	0. 034
Material	Haycite	m³	0. 007
	Solder joint	one	1. 000
	Root Irrigator (Φ 100mm × L915mm)	piece	1. 000
	Geotextile 250g	m²	0. 023
	Other material costs	Yuan	5. 560
Machinery	Other machinery costs	Yuan	0. 110

(2) Micro-Spraying and Thousand-Autumn Frame

Job responsibilities: Hole drilling, connecting quick-release nozzles, installing seven-piece spray head with support rods, and setting up swing frames.

Unit of measurement: set

Quota number			1-358	1-359	1-360	1-361
Project			Micro-spraying	Qianqiu frame installation		
				ϕ20	ϕ25	ϕ32
	Name	Unit	Quantity			
Man-made	Combined work day	Work day	0.010	0.020	0.025	0.029
Material	Insertion rod	one	1.000	–	–	–
	Quick fitting	one	1.000	–	–	–
	Micro-spraying 7-piece set	one	1.000	–	–	–
	Senkyu-kei 20	set	–	1.000	–	–
	Senkyu-kei 25	set	–	–	1.000	–
	Senkyu-kei 32	set	–	–	–	1.000
	Other material costs	yuan	0.210	1.940	2.240	2.440
Machin-ery	200L mortar mixer and	Tian-ban	1.000	–	–	–
	other machinery costs	Yuan	0.030	0.060	0.080	0.090

(3) Installation of drip irrigation, seepage irrigation, and sprinkler heads for three-dimensional flower beds

Job **content: on-site handling, appearance inspection, pipeline cutting, drilling, pipe installation, support rod installation, adjustable nozzle,**drip arrow component flower pot, flow stabilizer installation, etc. Unit of measurement: set

Quota number			1-362	1-363	1-364	1-365
Project			Surface adjustable nozzle installation	Installation of 3D Light Fog Nozzle	5-drip arrow group installation	16-drip arrow group installation
	Name	Unit	Quantity			
Man-made	Combined work day	Work day	0.055	0.026	0.049	0.098
Material	Adjustable nozzle	set	(1.000)	–	–	–
	UPVC hose ϕ4	set	–	0.515	1.020	0.515
	Direct-through ϕ4	set	–	1.010	1.010	1.010
	Clustered 16-drip arrow group	one	–	–	–	1.000
	Micro-spraying 7-piece set	one	–	1.000	–	–
	5Tong Dijian group	set	–	–	1.000	–
	Other material costs	yuan	–	0.190	0.090	0.460
Machin-ery	Other machinery costs	Yuan	0.170	0.080	0.150	0.310

Job responsibilities: On-site material handling, exterior inspection, pipeline trimming, drilling, conduit installation, support rod installation, and adjustable nozzle installation. The installation of the seepage system and the installation of the flow stabilizer are also included. measurement unit：m

Quota number			1−366	1−367
Project			Percolation system installation	Surface-adjustable nozzle PE connecting pipe
Name		Unit	Quantity	
Human being	Combined work day	Work day	0. 080	0. 022
Material	Percolation tube	m	1. 030	–
	Percolation tube blockage	one	2. 020	–
	PE pipe, Φ20	m	–	1. 020
	PE bypass Φ20	one	–	1. 010
	PE elbow, Φ20	one	–	1. 010
	UPVC hose Φ4	m	0. 618	–
	Direct-through Φ4	one	2. 020	–
	Stabilizer 10L	one	2. 000	–
	Nylon tie L=100~150	root	3. 030	–
	Other material costs	yuan	0. 140	0. 350
Mach-ine	Other machinery costs	Yuan	0. 250	0. 070

5. Oiling of Pipes and Iron Parts

Job duties: cleaning, rust removal, oil adjustment, and oil brushing. Unit: 10m

Quota number			1−368	1−369	1−370	1−371	1−372	1−373
Project			Second coat of red lead anti-rust paint on pipe, nominal diameter (within mm)					
			15	20	25	32	40	50
Name		Unit	Quantity					
Man-made	Combined work day	Work day	0. 135	0. 138	0. 173	0. 219	0. 249	0. 309
Material	Red lead anti-corrosive paint	kg	0. 231	0. 233	0. 291	0. 368	0. 418	0. 522
	Solvent gasoline 200#	kg	0. 055	0. 059	0. 074	0. 093	0. 101	0. 132
	Other material costs	Yuan	0. 060	0. 110	0. 070	0. 090	0. 100	0. 130
Machin-ery	Other machinery costs	Yuan	0. 420	0. 430	0. 540	0. 690	0. 780	0. 970

Job duties: cleaning, rust removal, oil adjustment, and oil brushing. Unit: 10m

Quota number			1-374	1-375	1-376
Project			Second coat of red lead anti-rust paint on pipe, nominal diameter (within mm)		
			70	80	100
Name		Unit	Quantity		
Man-made	Combined work day	Work day	0. 390	0. 442	0. 588
Material	Red lead anti-corrosive paint	kg	0. 657	0. 770	0. 992
	Solvent gasoline 200#	kg	0. 166	0. 195	0. 251
	Other material costs	Yuan	0. 160	0. 190	0. 240
Machin-ery	Other machinery costs	Yuan	1. 230	1. 340	1. 850

Job duties: cleaning, rust removal, oil adjustment, and oil brushing. Unit: 10m

Quota number			1-377	1-378	1-379	1-380	1-381	1-382
Project			Nominal diameter (within mm) for second coat of asphalt paint on pipe					
			15	20	25	32	40	50
Name		Unit	Quantity					
Man-made	Combined work day	Work day	0. 137	0. 138	0. 173	0. 219	0. 249	0. 309
Material	Asphalt paint	kg	0. 446	0. 449	0. 563	0. 710	0. 807	1. 008
	Motor benzene	kg	0. 072	0. 073	0. 092	0. 115	0. 131	0. 164
	Other material costs	Yuan	0. 100	0. 100	0. 130	0. 160	0. 180	0. 230
Machin-ery	Other machinery costs	Yuan	0. 430	0. 430	0. 540	0. 690	0. 780	0. 970

Job duties: cleaning, rust removal, oil adjustment, and oil brushing. Unit: 10m

Quota number			1-383	1-384	1-385
Project			Nominal diameter (within mm) for second coat of asphalt paint on pipe		
			70	80	100
Name		Unit	Quantity		
Man-made	Combined work day	Work day	0. 390	0. 457	0. 588
Material	Asphalt paint	kg	1. 269	1. 487	1. 916
	Motor benzene	kg	0. 206	0. 242	0. 312
	Other material costs	Yuan	0. 290	0. 340	0. 440
Machin-ery	Other machinery costs	Yuan	1. 230	1. 440	1. 850

Job duties: cleaning, rust removal, oil adjustment, and oil brushing. Unit of measurement: 10kg

Quota number			1-386
Project			Red Dan Anti-Rust Paint Second Coat on Iron Parts
Name		Unit	Quantity
Man-made	Combined work day	Work day	0. 065
Material	Red lead anti-corrosive paint	kg	0. 211
	Solvent gasoline 200#	kg	0. 056
	Other material costs	Yuan	0. 050
Machin-ery	Other machinery costs	Yuan	0. 200

6. Well Body Masonry

Job responsibilities: Material transportation, mortar application, brick delivery, bricklaying, and manhole cover installation. Unit of measurement: see table

Quota number			1-387	1-388	1-389
Project			Green space water supply well		Manhole cover installation
			Circular (Within 1.2 m depth)	Rectangular valve box well (Within 1.2 m depth)	
			Seat		Cover
Name		Unit	Quantity		
Man-made	Combined work day	Work day	1.674	1.013	0.574
Material	Cement mortar | M5.0-S-3	m³	0.289	0.122	–
	Cast iron manhole cover seat	set	–	–	1.000
	Plank material	m³	–	–	0.008
	Shale brick	piece	485.000	268.000	–
	Valve box VB-1320	one	–	1.000	–
	Ready-mixed concrete C10	m3	–	–	0.107
	Other material costs	yuan	5.080	7.190	5.020
Machin-ery	Other machinery costs	Yuan	5.270	3.190	1.810

7. Installation of Automatic Intelligent Sprinkler Irrigation System

(1) Sensing Device

Job responsibilities: pole erection, installation, commissioning, etc. Unit of measurement: set

Quota number			1-390	1-391	1-392
Project			Wired		Wireless
			Switching mode	Smart	
Name		Unit	Quantity		
Man-made	Combined work day	Work day	1.410	2.810	1.410
Material	Acceptor	one	–	–	(1.000)
	Telecontroller	one	–	–	(1.000)
	Sensing device	set	(1.000)	(1.000)	–
	Upright	m	(3.000)	(3.500)	–
	Pre-embedded (welded) iron parts	kg	16.034	16.034	–
	Low-carbon hot-rolled wire rod (high wire) HPB235 with diameter up to Φ10	kg	4.382	4.382	–
	Hot-rolled smooth round steel bar HPB235 with diameter ≥Φ10	kg	9.622	9.622	–
	M16×65~80 nut bolt	set	4.120	4.120	
	receiver clip	one	–	–	1.000
	Water joint	one	–	–	2.000
	Electricity	kw·h	1.000	1.000	–
	Ready-mixed concrete C15	m³	0.066	0.066	
	Ready-mixed concrete C25	m³	0.222	0.222	–
	Other material costs	yuan	3.540	3.540	1.490
Machin-ery	Other machinery costs	Yuan	4.440	8.850	4.440

(2) Controller, Module

Job responsibilities: Installation and fixation, drilling, wiring, foundation construction, electrical wiring, grounding, etc. Unit of measurement: unit

Quota number			1-393	1-394	1-395	1-396	1-397	1-398
Project			Wired controller					
			Wall mounting type			Console mode		
			Specifications (Station)					
			4	6	8	4	6	8
Name		Unit	Quantity					
Man-made	Combined work day	Work day	1. 850	2. 000	2. 000	2. 145	2. 000	2. 200
Material	Controller	Block	(1. 000)	(1. 000)	(1. 000)	(1. 000)	(1. 000)	(1. 000)
	Shale brick	Block	–	–	–	62. 830	79. 310	79. 310
	Stent expansion φ8	Block	4. 000	4. 000	4. 000	–	–	–
	Stent Φ12	Block	–	–	–	4. 000	4. 000	4. 000
	Wiring clamp	Block	2. 000	2. 000	2. 000	–	–	–
	Ready-mixed concrete C20	m^3	–	–	–	0. 124	0. 155	0. 155
	Other material costs	yuan	0. 680	0. 680	0. 730	1. 820	2. 140	2. 190
Machinery	Other machinery costs	Yuan	5. 820	6. 300	6. 930	6. 750	6. 890	6. 920

Job responsibilities: Installation and fixation, drilling, wiring, foundation construction, electrical wiring, grounding, etc. **Unit of measurement: unit**

Quota number			1-399
Project			Controller
			Wireless
Name		Unit	Number **Quantity**
Man-made	Combined work day	Work day	0. 169
Material	Acceptor	ONE	(1. 000)
	Controller	Block	(1. 000)
	Telecontroller	one	(1. 000)
	Dry cell	one	1. 000
	Other material costs	yuan	1. 150
Machinery	Other machinery costs	Yuan	0. 530

Job responsibilities: Installation and fixation, drilling, wiring, foundation construction, electrical wiring, grounding, etc. Unit of measurement: unit

Quota number			1-400	1-401	1-402
Project			Module		
			Specifications (Station)		
			4	6	8
Name		Unit	Quantity		
Man-made	Combined work day	Work day	0.165	0.242	0.330
Material	Module	Set	(1.000)	(1.000)	(1.000)
	Other material costs	Yuan	0.510	0.510	0.560
Machin-ery	Other machinery costs	Yuan	0.520	0.760	1.040

(3) Decoder

Job responsibilities: Installation, wiring, and commissioning. Unit of measurement: pieces

Quota number			1-403	1-404	1-405	1-406
Project			Specifications (Station)			
			1	2	4	6
Name		Unit	Quantity			
Man-made	Combined work day	Work day	0.363	0.473	0.616	0.798
Material	Decoder	Set	(1.000)	(1.000)	(1.000)	(1.000)
	Wiring clamp	one	1.020	1.020	1.020	1.020
	Waterproof elbow 32	one	2.000	4.000	8.000	12.000
	Other material costs	yuan	0.750	0.990	1.410	1.890
Machin-ery	Other machinery costs	Yuan	1.140	1.490	1.940	2.510

(4) Grounding Electrophoresis Protector

Job responsibilities: Installation, wiring, and commissioning. Unit of measurement: group

Quota number			1-407	1-408
Project			Grounding electrophoresis protector	
			Wiring	Grounding rod
Name		Unit	Quantity	
Man-made	Combined work day	Work day	1.238	1.238
Material	Grounding rod	root	(3.000)	(1.000)
	Copper rod clamp	one	(4.000)	(1.020)
	Lightning arrester	one	(1.000)	(1.000)
	$6mm^2$ of bare copper wire	m	18.000	6.000
	Other material costs	yuan	1.700	1.750
Machin-ery	Other machinery costs	Yuan	3.900	3.900

8. Fixed Rate Fertilization System Installation

Job responsibilities: On-site material handling, visual inspection, installation and commissioning of fertilization systems, etc. Unit of measurement: set

Quota number			1-409	1-410
Project			Fixed rate fertilization system installation	
			3t	10t
Name		Unit	Quantity	
Man-made	Combined work day	Work day	0.800	0.800
Material	Fixed-rate fertilization system 3t	Set	(1.000)	–
	Fixed-rate fertilization system 10t	Set	–	(1.000)
	Other material costs	Yuan	1.520	2.540
Machin-ery	Other machinery costs	Yuan	2.520	2.520

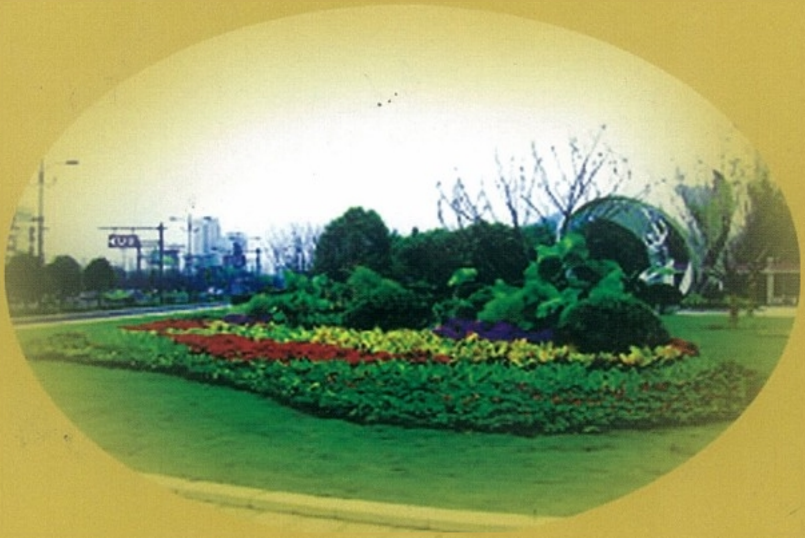

0 001551 60553 >

Unified Book Number: 155160 ·553
Price: 160.00 yuan
